# Canonical and noncanonical roles of Hop1 are crucial for meiotic prophase in the fungus *Sordaria macrospora*

**DOI:** 10.1371/journal.pbio.3002705

**Published:** 2024-07-01

**Authors:** Emeline Dubois, Stéphanie Boisnard, Henri-Marc Bourbon, Kenza Yefsah, Karine Budin, Robert Debuchy, Liangran Zhang, Nancy Kleckner, Denise Zickler, Eric Espagne

**Affiliations:** 1 Université Paris-Saclay, Commissariat à l’Énergie Atomique et aux Énergies Alternatives (CEA), Centre National de la Recherche Scientifique (CNRS), Institute for Integrative Biology of the Cell (I2BC), Gif-sur-Yvette, France; 2 Centre de Biologie Intégrative, Molecular, Cellular & Developmental Biology Unit, Université Fédérale de Toulouse, Toulouse, France; 3 Center for Reproductive Medicine, Cheeloo College of Medicine, Shandong University, Jinan, Shandong, China; 4 Department of Molecular and Cellular Biology, Harvard University, Cambridge, Massachusetts, United States of America; Johns Hopkins University, UNITED STATES OF AMERICA

## Abstract

We show here that in the fungus *Sordaria macrospora*, the meiosis-specific HORMA-domain protein Hop1 is not essential for the basic early events of chromosome axis development, recombination initiation, or recombination-mediated homolog coalignment/pairing. In striking contrast, Hop1 plays a critical role at the leptotene/zygotene transition which is defined by transition from pairing to synaptonemal complex (SC) formation. During this transition, Hop1 is required for maintenance of normal axis structure, formation of SC from telomere to telomere, and development of recombination foci. These *hop1Δ* mutant defects are DSB dependent and require Sme4/Zip1-mediated progression of the interhomolog interaction program, potentially via a pre-SC role. The same phenotype occurs not only in *hop1Δ* but also in absence of the cohesin Rec8 and in *spo76-1*, a non-null mutant of cohesin-associated Spo76/Pds5. Thus, Hop1 and cohesins collaborate at this crucial step of meiotic prophase. In addition, analysis of 4 non-null mutants that lack this transition defect reveals that Hop1 also plays important roles in modulation of axis length, homolog-axis juxtaposition, interlock resolution, and spreading of the crossover interference signal. Finally, unexpected variations in crossover density point to the existence of effects that both enhance and limit crossover formation. Links to previously described roles of the protein in other organisms are discussed.

## Abstract 2

Hop1 has been previously shown to display diverse meiotic functions in different organisms. This study identifies and characterizes the functions of Hop1 in the fungus Sordaria macrospora and shows that it plays a crucial role during the leptotene/zygotene transition and formation of the synaptonemal complex during meiotic prophase.

## Introduction

During the meiotic program of most organisms, recombination at the DNA level, recombination-mediated pairing of maternal and paternal chromosomes (homologs), as well as progressive organization of higher-order structure along chromosomes, all occur in direct functional linkage. This linkage is mediated in large part via direct physical association of recombination complexes with chromosome axes that comprise: meiotic and mitotic cohesin complexes, cohesin-associated proteins like Pds5/Spo76 and Wapl, condensins, Topoisomerase II and meiosis-specific axis proteins. The latter include one or several HORMA domain-containing proteins (budding-yeast Hop1, mammalian HORMAD1 and HORMAD2, plant ASY1/PAIR2 and ASY2, and *Caenorhabditis elegans* HIM-3, HTP-1, HTP-2 and HTP-3) and a coiled-coil domain protein (budding-yeast Red1, mammalian SYCP2/SYCP3 and plant ASY3/PAIR3/DSY2 and ASY4) that binds directly to Hop1 in vitro and in cells (review and references in [1–3]). Many gaps still exist in our understanding of exactly how axis structure arises, what are the natures of the molecular and functional interactions among axis components and of axes with their associated recombination complexes (e.g. [2,4–7], review in [3,8]).

In the current work, we have exploited the power of the fungus *Sordaria macrospora* as a system for visualizing whole chromosome dynamics in relationship with the recombination process to further investigate the role(s) of this organism’s HORMA-domain component Hop1 during meiotic prophase. Previous studies in other organisms indicate that this protein presents diverse patterns of localization throughout synapsis. Hop1 is also involved differently among different organisms with respect to key meiotic events like formation of DNA double-strand breaks (DSBs), crossovers, and synaptonemal complex (SC) (review and references in [1–3,8]). For example, in budding yeast, mouse, and *C*. *elegans*, Hop1 is required for initiation of recombination while, in *Arabidopsis thaliana*, DSB formation is Hop1-independent [9–12]. Our current findings add to this diversity, as described below.

The present study includes identification of *Sordaria* Hop1 protein and documentation of its chromosomal loading patterns in wild type and diverse pairing and recombination mutants. Analysis of a complete *HOP1* deletion mutation and of 6 mutant alleles that express different portions or mutated forms of the Hop1 protein, allowed detailed elucidation of multiple component roles of Hop1 in: (i) loading of the protein; (ii) modulation of axis length; (iii) resolution of chromosome entanglements formed during pairing; and (iv) crossover patterning and interference. We found also that *Sordaria* Hop1 is specifically required during the transition from homolog coalignment to synapsis for the maintenance of the cohesin-associated protein Spo76/Pds5 (but not for maintenance of the cohesin Rec8) along chromosome axes. Maintenance of Spo76/Pds5 along the chromosomes is correlated with initiation of the SC because its axis localization is maintained in absence of recombination and Sme4/Zip1 and thus SC formation. Mutations in *REC8* and *SPO76/PDS5* confer analogous effects. These 3 axis-associated molecules are thus required collaboratively to ensure regular progression from leptotene to pachytene. Implications of these and other findings as well as relationships to Hop1 roles in other organisms are discussed.

## Results

### Identification, structure, and expression of *Sordaria macrospora* Hop1

*1*. *Identification and phylogeny*. *Sordaria macrospora HOP1* (SMAC_06964) was identified by a series of phylogenomics-oriented homology searches for Hop1, coupled to multiple sequence alignments of fungal genomes. As for budding and fission yeasts [13,14], the genome of *S*. *macrospora* contains a single *HOP1* gene, in contrast to plants and mammals that contain 2 paralogs (ASY1/PAIR2 and ASY2; HORMAD1 and HORMAD2, respectively) and *C*. *elegans* which contains 4 paralogs: HIM-3, HTP-1, HTP-2, and HTP-3 [9,12,15–21].

A phylogenic tree analysis (by IQTREE; http://iqtree.cibiv.univie.ac.at/) shows that SMAC_06964 is a bona fide Hop1 homolog. In addition to Hop1, as for most eukaryotes, the *S*. *macrospora* genome contains also 2 other HORMA domain proteins: the subunit of DNA polymerase Zeta Rev7 (SMAC_04698) and the spindle-checkpoint protein Mad2 (SMAC_09019) (S1A Fig).

**Fig 1:**
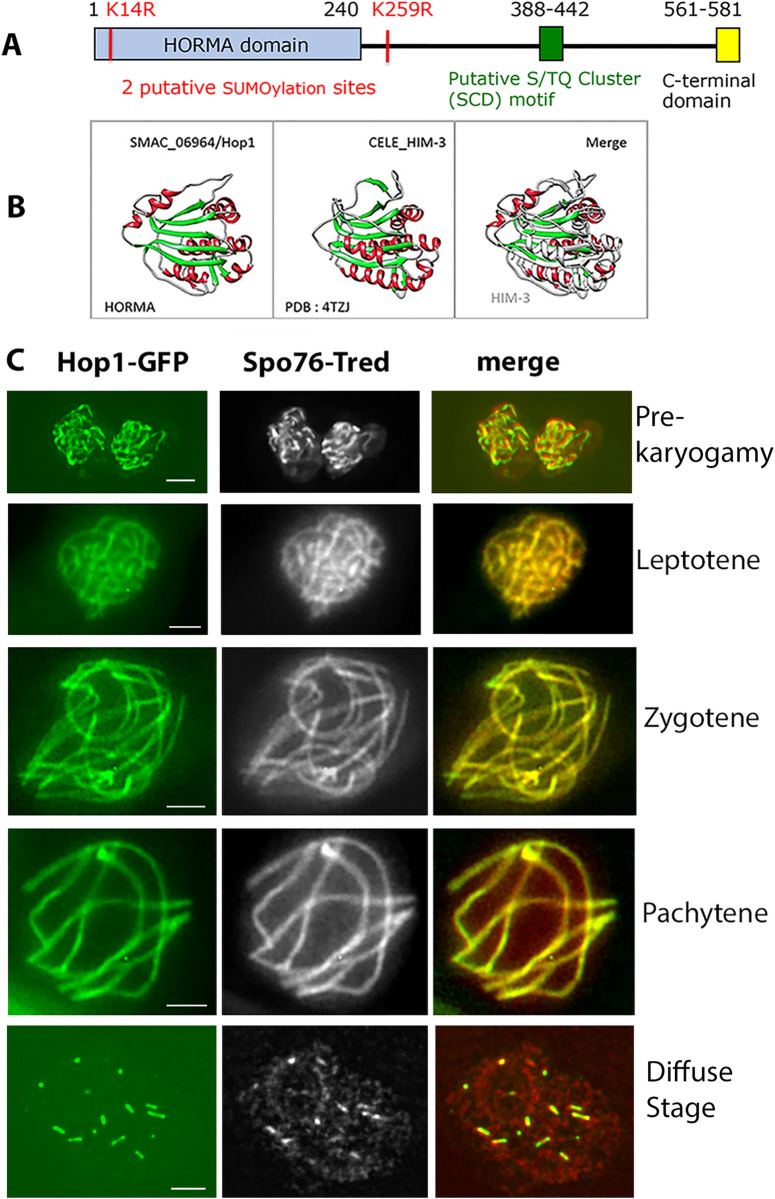
Schematic domain organization, structure, and localization of Hop1 in WT. (**A**) Schematic domain organization of *Sordaria* Hop1. (**B**) AlphaFold2 modelled *Sordaria* Hop1 HORMA domain (left), atomic (PDB:4TZJ), and experimentally determined *C*. *elegans* HIM-3 (middle) share structural similarities as shown by their superimposition (HIM-3 gray, right). Alpha-helices and beta-strands are in red and green, respectively. (**C**) Localization of Hop1 in WT meiosis. Hop1-GFP (left) and Spo76/Pds5-TdTomato (Tred, middle) are perfectly colocalized in the 2 pre-karyogamy haploid nuclei (top) and from leptotene to the post-pachytene diffuse stage (bottom). Scale bars: 2 μm. SCD, S/TJQ cluster domain; WT, wild-type.

*2*. *Structure*. Schematic domain organization of the predicted 581 amino acid of the *Sordaria* Hop1 protein is shown in Fig 1A. Analysis of Hop1 domains using the SMART software (http://smart.embl-heidelberg.de/), coupled with multiple sequence alignments of fungal and other orthologs, shows that *Sordaria* Hop1 contains conserved domains also described for the other Hop1 orthologs: an N-terminal HORMA domain, a conserved SCD (S/TJQ cluster domain), and 2 putative SUMOylation sites (e.g., [6,22]). In contrast, the C-terminal domain (CTD; which can mediate and allow modulation of interactions with partner proteins as well as self-oligomerization) shows no clear similarity with the closure motif (CM), described for example in budding yeast and *A*. *thaliana* (e.g., [3,23]). A clear CM is also absent in Hop1 of the close relative fungus *Neurospora crassa* [24], which shows 80% identity with the *Sordaria* Hop1 protein. Moreover, despite conservation among many organisms, *S*. *macrospora* and *N*. *crassa* Hop1, also lack an identifiable central chromatin-binding region (Fig 5A in [24]).

Modelling with AlphaFold2 (AF2) (see S1B Fig for confidence values of the predicted 3D model) coupled to a structural similarity search among experimentally determined atomic structures within the Protein Data Bank archive, revealed that the HORMA domain of *Sordaria* SMAC_06964 is most homologous to the *C*. *elegans* HIM-3 HORMA domain (Fig 1B), with a root mean square deviation (RMSD) value of 2.4 Angstroms over 146 Ca atoms. Taken together, our phylogenetic and structural data establish that SMAC_06964 is Hop1.

*3*. *Expression of Sordaria Hop1 is specific to the sexual cycle*. Kinetics of Hop1 were obtained by comparison of RT-qPCR analyses of meiosis versus vegetative cells. Hop1 transcripts are specifically induced during the sexual cycle (initiation at day 2 in S2A Fig) and remain at approximately the same level through the meiotic divisions. Their kinetics parallel, although at a higher level, those of transcripts for the meiotic trans-esterase Spo11 and the axis-localized Asy2/Mer2 protein (S2A Fig), both of which are required for DSB formation in *Sordaria* as in other organisms (e.g., [25]).

*Sordaria* Hop1 localizes to chromosome axes from S-phase to the end of pachytene

Hop1 localization to chromosomes in wild type (WT) and in mutants of interest was examined by single cell imaging using a *HOP1-GFP* C-terminal fusion (expressed under the *HOP1* promoter control), which replaces completely the resident *HOP1* gene. The corresponding protein as well as the protein produced by the *HOP1-mCherry* gene introduced ectopically in the genome is perfectly functional: when introduced in *hop1Δ*, all fruiting bodies produce 90% eight-spored asci alike the WT strain (S2B Fig, right). Moreover, in *Sordaria*, progression of meiosis can be monitored by progressive increase in ascus (meiocyte) size and an accompanying increase in nuclear volume (S2C Fig, left and middle). Since both increases occur irrespective of the state/progression of chromosomal events, they provide a “clock” for the meiotic process, against which the timing of mutant chromosomal events can be compared. Accordingly, all comparisons, including counts of SC segments and/or foci were appreciated by comparison of asci with similar lengths in WT and mutants. The *hop1* mutants do not alter the ascus length clock (example of *hop1Δ* in S2C Fig, right).

In the WT strain, Hop1 is only observed on chromosomes during meiosis where it is a major component of chromosome axes from leptotene to end pachytene, as indicated by its perfect co-localization with the cohesin-associated Spo76/Pds5 protein (Fig 1C). Hop1 first appears during premeiotic S-phase (in the non-yet-fused haploid nuclei) as very short lines concomitant with lines of the axis component Spo76/Pds5 (Fig 1C, top). The Hop1 signal becomes increasingly continuous during early leptotene and runs the full length of chromosomes as smooth continuous lines from coalignment throughout late pachytene (Fig 1C). Like Spo76/Pds5, Hop1 is progressively removed as the homologs desynapse until, by the diffuse stage, it is no longer detectable except in the few remaining SC segments (Fig 1C, bottom). We note that this localization contrasts with that in budding yeast, mouse, and plants, where the level of Hop1 orthologs decreases significantly as chromosomes progress from zygotene to pachytene [5,12,15–17,26,27].

### Axis association of Hop1 does not require DSBs or recombination proteins

**Fig 2:**
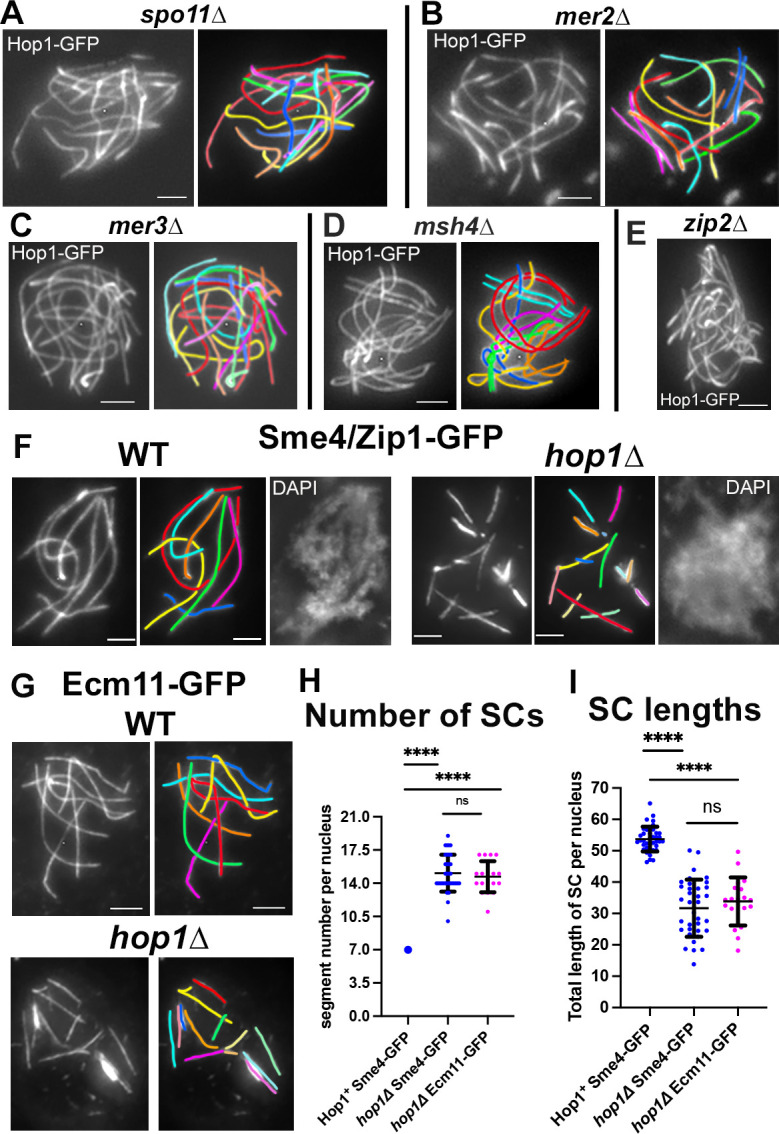
Hop1 localizes along axes in recombination mutants but synapsis is defective when absent. (**A**, **B**) Hop1 localizes all along the 14 unsynapsed chromosome axes in absence of DSBs in *spo11Δ* (**A**) and *mer2Δ* mutants (**B**) as confirmed by corresponding drawings. (**C**–**E**) Same localization of Hop1-GFP in *mer3Δ* (**C**), *msh4Δ* (**D**), and *zip2Δ* (**E**) mutants with defective coalignment. (**F**) Sme4/Zip1-GFP localization in WT and *hop1Δ* with corresponding drawings (middle) and DAPI (right). SC installation occurs in both WT (left) and *hop1Δ* (right) but instead of the 7 SCs seen in WT (left), 14 segments of SCs are seen in the mutant (right). (**G**) Similar contrast in SC formation between WT (top) and *hop1Δ* (bottom) observed with the SC central-component Ecm11. Scale bars: 2 μm. (**H**) Comparison of the distribution of the number of Sme4 (blue) and Ecm11 (magenta) SC segments in *hop1Δ* vs. WT SCs (large blue dot). (**I**) Comparison of the SC lengths between WT (left) and *hop1Δ* (right). (**H, I**): *n* = 39, 35, and 19 nuclei, respectively. Mean and error bar (SD) are indicated for each set. Statistical significance was assessed using two-tailed Student’s *t* test; ns, not significant (*P* ≥ 0.05); ****P < 0.0001. The raw data underlying panels 2H and 2I are available in S1 Data. DSB, double-strand break; SC, synaptonemal complex; WT, wild-type.

Hop1 localization was first analyzed in 2 mutants *spo11Δ* and *mer2Δ* which do not form DSBs and remain thus asynaptic until end pachytene [25]. The haploid number of chromosomes being 7 in *Sordaria*, Hop1 is visible along the axes of the 14 un-synapsed chromosomes (shown by different colors) in both mutants from leptotene throughout pachytene indicating that Hop1 localization on axis is independent of both of these molecules and of the DSB formation itself (Fig 2A and 2B; *n* = 20 nuclei for each mutant). Secondly, Hop1 localizes also normally in absence of post-DSB recombination proteins in *mer3Δ* and *msh4Δ* (Fig 2C and 2D; *n* = 20 nuclei for each mutant) which make DSBs but exhibit defects in later stages as manifested in aberrant coalignment and synapsis (illustrated in Fig 2D by un-synapsed yellow and blue homologs compared to perfectly coaligned red and cyan homolog). Thirdly, Hop1 localization is also independent of the Zip2-Zip4 complex (Fig 2E) which plays a central role in SC nucleation [28]. In accord with normal axis formation in these mutants, they all exhibit mean WT-like axis lengths as measured by Hop1-GFP (52.8 ± 3 microns in *spo11Δ* versus 53.7 ± 4 microns in WT; *n* = 20 and 150 nuclei, respectively; ± indicates the standard deviation, here and below).

### In the absence of Hop1, the synaptonemal complex initiates normally at zygotene but is never complete from telomere to telomere

In *hop1Δ*, as in WT, SC installation (observed by the SC transverse filament Sme4/Zip1-GFP) initiates at zygotene in several small nucleation sites (S3A Fig and below). However, at pachytene, in contrast to WT where SCs are formed along all the seven homologs (Fig 2F, left), *hop1*Δ nuclei exhibit only short segments of SCs (Fig 2F, right). The same contrast between WT and mutant is also observed with the SC central-region protein Ecm11 (Fig 2G, top and bottom, respectively), implying that the defect pertains to the entire tripartite SC structure. As expected, the number of Sme4 and Ecm11 segments is significantly similar: two-tailed Student’s test; *P* ≥ 0.05; Fig 2H; details in corresponding legend. The total per-nucleus length of *hop1Δ* SC segments is significantly different from WT (60% of the full WT SC length: 31.7 ± 9.1 (Sme4) and 33.8 ± 7.6 (Ecm11) microns, versus 53.7 ± 4 microns in WT, but again significantly similar (*P* ≥ 0.05) for the 2 proteins (Fig 2I).

Although incomplete, SC formation in the *hop1Δ* mutant exhibits the same kinetics and functional dependencies as in WT: (i) SCs appear at zygotene as small Sme4/Zip1-GFP segments (S3A Fig, left versus right and see below) and disappear progressively as the homologs desynapse until, by the diffuse stage, they are no longer detectable except in a few remaining segments (illustrated by Ecm11-GFP + Hei10-GFP, S3B Fig). (ii) SC formation is DSB dependent: like in the single *spo11Δ* mutant, only univalents are visible in the *hop1Δ spo11Δ* double mutant (S3C Fig, left and right, respectively; *n* = 15 and 20 nuclei). (iii) Also, just as in the *sme4Δ* single mutant, homologs are coaligned at 200 nm distance in the *hop1Δ sme4Δ* double mutant, but do never progress beyond this stage and, thus, do not form SCs (S3D Fig, left and right, respectively; *n* = 40 and 20 nuclei).

### In the absence of Hop1, Spo76/Pds5 is lost specifically in the subset of chromosomal regions that lack SC

To further define the basis for absence of SCs in some chromosome regions of *hop1*Δ, we examined nuclei where chromosome axes were marked by Spo76/Pds5. This protein is a component of the cohesin complex, which in turn, is known to interact with and/or occur in close spatial proximity to Hop1 (e.g., [4]; review in [3,5,8]).

**Fig 3:**
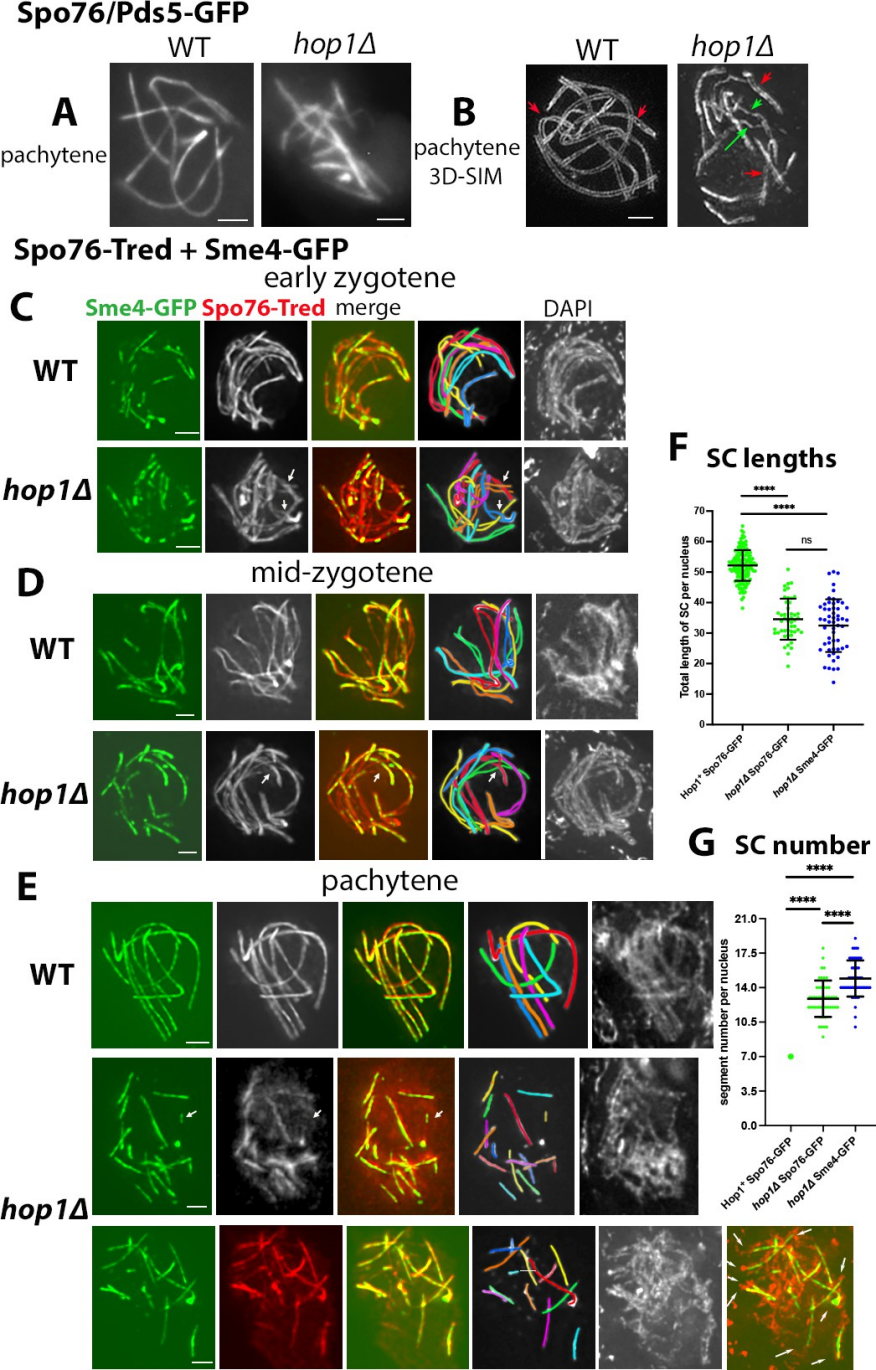
Synapsis pattern of *hop1Δ* observed with Spo76 and Sme4/Zip1. (**A**, **B**) Spo76-GFP localization during pachytene in WT (left) and in absence of Hop1 (right) in fluorescent microscopy (**A**) and in 3D-SIM (**B**). Red arrows in (**B**) point to regular homolog spacing in both WT and *hop1Δ*; green arrows (right) indicate regions with axis separation. (**C–E**) Colocalization of Sme4/Zip1-GFP (left) and Spo76-Tred (second from left in black and white) with corresponding merge plus drawing of the 7 homologs (with different colors, middle) in WT (top) and *hop1Δ* (bottom); right: corresponding DAPI. (**C, D**) At early zygotene (**C**), SCs initiate preferentially at chromosome ends and elongate during mid zygotene (**D**) in both strains. Note that Spo76-Tred is visible on chromosome axes in both unsynapsed and synapsed chromosome regions. Arrows in the mutant nuclei point to interlockings. (**E**) Pachytene. In the WT nucleus (top) Sme4 forms 7 continuous lines, while in the 2 *hop1Δ* nuclei (bottom) only segments of Sme4/SCs are formed. Note also that Spo76-Tred colocalizes with Sme4 along all homologs in WT, but is only visible in the synapsed regions in the mutant. Arrow in the top *hop1Δ* nucleus points to different staining of Sme4 and Spo76 in a small SC stretch. In the bottom *hop1Δ* nucleus (right), merge of Sme4/Spo76 (orange) with DAPI (red) indicates that SC segments are mostly located to chromosome ends (arrows). Scale bars: 2 μm. (**F, G**) Quantification of lengths and number of Spo76-GFP (green) and Sme4-GFP (blue) segments seen in WT and *hop1Δ* nuclei. Statistical significance was assessed by a two-tailed Student’s *t* test; ns, not significant (*P* ≥ 0.05); ****P < 0.0001. Mean and error bar (SD) are indicated for each set. (**F**) The lengths of the *hop1Δ* pachytene Spo76 and Sme4 segments are not significantly different, which confirms that Spo76 is maintained only in synapsed regions. (**G**) The WT strain exhibits always 7 Spo76/Sme4 colocalized segments (large green dot) while the number of Spo76 and Sme4 stretches (12.9 +/− 1.9 and 15.6 +/− 1.9) are significantly different. Number of measured nuclei: *n* = 145, 47, and 54, respectively. The raw data underlying panels 3F and 3G are available in S1 Data. 3D-SIM, three-dimensional structured illumination microscopy; SC, synaptonemal complex; WT, wild-type.

Throughout pachytene, instead of the 7 Spo76-GFP lines seen in WT (Fig 3A, left), *hop1Δ* nuclei exhibit always more than 7 Spo76-GFP segments (Fig 3A, right). This phenotype is confirmed by three-dimensional structured illumination microscopy (3D-SIM) microscopy. In WT, synapsis occurs regularly along the 7 *Sordaria* homologs, with Spo76-GFP axes separated by a constant distance all along the homolog lengths (red arrows in Fig 3B, left). In contrast, *hop1Δ* nuclei exhibit mostly short segments, wherein Spo76-GFP axes show the same parallel relationship seen in WT, implying the presence of an SC (red arrows in Fig 3B, right). Thus, in *hop1Δ*, Spo76 is retained or lost in concert with progression/absence of SC. Occasionally, Spo76-GFP also marks more widely open axes, indicating regions where synapsis has not occurred (green arrows in Fig 3B, right).

To confirm and extend these conclusions, we compared the co-localization of Spo76-Tred and Sme4/Zip1-GFP in both WT and *hop1Δ* from early zygotene on (Fig 3C–3E). The first 2 stages of zygotene are similar in the 2 strains: Sme4 occurs in short stretches at early zygotene (Fig 3C) and in longer stretches at mid-zygotene (Fig 3D). Also, during both stages, Spo76-Tred shows continuous lines along all chromosomes (Fig 3C and 3D and corresponding drawings). It is also notable that SC initiates preferentially at chromosome ends (as shown by corresponding DAPI). Hop1 is, thus, not required for SC initiation and elongation, as manifested in zygotene nuclei. Interestingly, all 20 analyzed zygotene *hop1Δ* nuclei contain at least 1 interlocking (arrows in Fig 3C and 3D), whereas the 20 corresponding WT nuclei show only 1 entanglement.

However, by pachytene, *hop1Δ* nuclei (Fig 3E) lack the continuous threadlike pattern of WT Sme4/SCs (Fig 3E, top; *n* = 40 nuclei). Furthermore, it is clear that Spo76/Pds5 remains visible (and is thus maintained) in all Sme4 SC segments but is no longer visible in the chromosome regions that lack an SC (Fig 3E and more examples in S3E Fig, left and right). Hop1 is thus required not only for SC progression beyond mid-zygotene, but also for the maintenance of Spo76/Pds5 along non-synapsed chromosome axes during pachytene.

Consistent with the observations above (Fig 2F and 2G), the lengths of the colocalized Spo76/Sme4 lines are significantly different from WT, but not significantly different from the SC lengths found by analysis of the Sme4 and Ecm11 segments alone (two-tailed Student’s *t* test; *P* ≥ 0.05; Fig 3F). When compared to WT (7 lines; green dot in Fig 3G), the number of Spo76 lines and Sme4 stretches (12.9 ± 1.9 and 15.6 ± 1.9) are significantly different (Fig 3G) but this difference can be explained by the fact that Spo76-Tred is always visible in the longer Sme4 stretches but is sometimes absent in the smaller stretches (arrow in Fig 3E). Additionally, in the early *hop1Δ* pachytene nuclei, when DAPI is less diffuse than at mid-late pachytene, chromosome ends can be identified (arrows in Fig 3E, bottom, right). Colocalization of Sme4 with DAPI shows that 9 of the 13 ends (the 14th end is attached to the nucleolus) contain an SC segment. This phenotype suggests that while SC initiates normally, preferentially at chromosome ends, it fails to fully elongate in the mutant. It shows also that Sme4/Spo76 segments are localized between the 2 homologs.

We also find that the Spo76 defect is strictly DSB and Sme4/Zip1 dependent because Spo76 is not lost from the chromosome axes in either *hop1Δ spo11Δ* or *hop1Δ sme4* double mutants (S3C and S3D Fig).

### *rec8Δ* and the *spo76-1* mutant show the same aberrant pachytene phenotypes as *hop1Δ*

**Fig 4:**
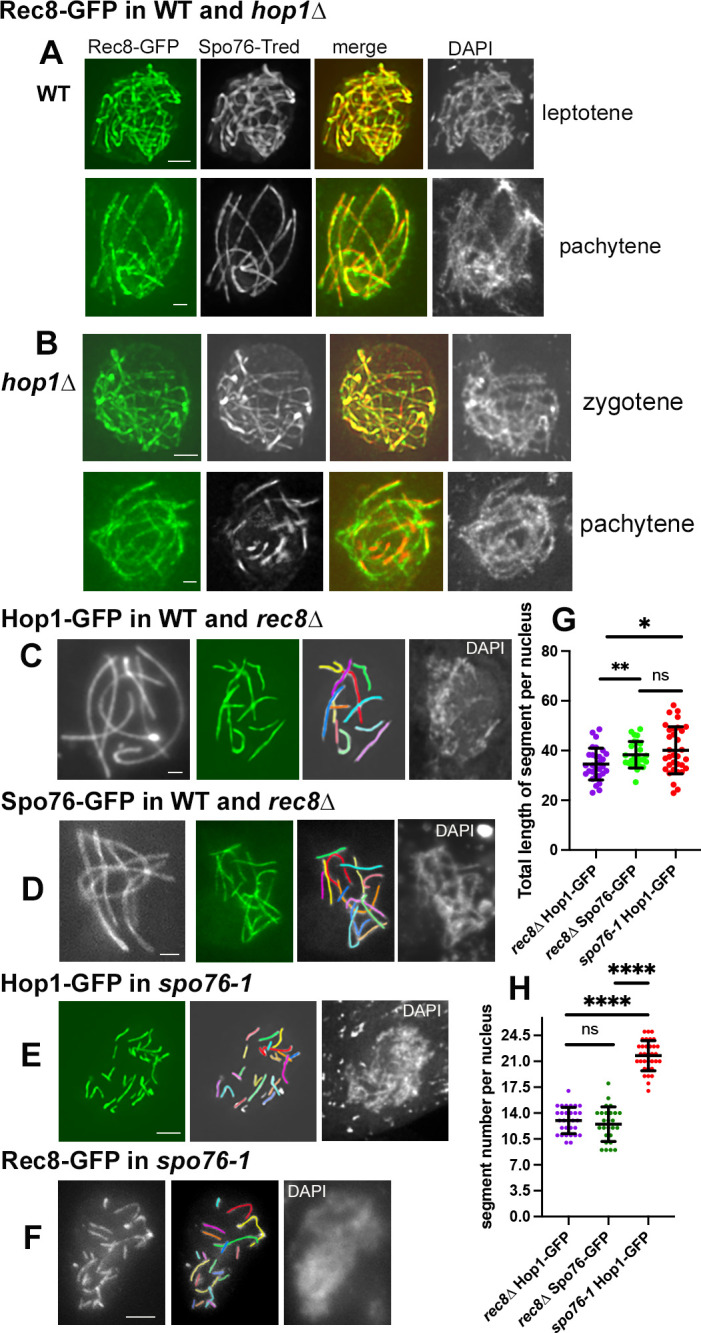
Reciprocal localization of Rec8, Spo76/Pds5, and Hop1 in corresponding mutants. (**A**, **B**) Colocalization of Rec8-GFP (left) and Spo76-Tred (second from left, black and white) with corresponding merge and DAPI (right). (**A**) In WT, both proteins colocalize perfectly during leptotene (top) and pachytene (bottom). (**B**) Both proteins colocalize also perfectly during *hop1Δ* leptotene (see S3F Fig) and zygotene (top), but only partially at pachytene (bottom). (**C, D**) WT left (black and white) and mutant right with corresponding drawing and DAPI (right). In absence of Rec8, the continuous localizations of Hop1-GFP (**C**) and Spo76-GFP (**D**) along the 7 WT bivalents (left) are disrupted in the mutant (middle to right). (**E, F**) In *spo76-1*, both Hop1-GFP (**E**) and Rec8-GFP (**F**) exhibit discontinuous lines during pachytene; right: corresponding DAPI. Scale bars: 2 μm. (**G, H**) Comparison of total segment lengths (**G**) and number of segments (**H**) per pachytene nucleus in *rec8Δ* nuclei using Hop1-GFP (purple) or Spo76-GFP (green) as axis marker; pachytene segments of Hop1-GFP (red) in *spo76-1*. Statistical significance was assessed using two-tailed Student’s *t* test; ns, not significant (*P* ≥ 0.05); *P < 0.05; **P < 0.01; ****P < 0.001. From left to right, *n* = 30, 26, and 33 nuclei, respectively. Mean and error bar (SD) are indicated for each set. The raw data underlying panels 4G and 4H are available in S1 Data. WT, wild-type.

We next tested whether other structural components of the meiotic chromosomes like the meiotic kleisin Rec8 regulate SC progression. Contrary to Spo76/Pds5, Rec8 is not lost from the chromosome axes in *hop1Δ*. In WT meiosis, with respect to chromosome axes, the temporal patterns of Rec8 localization during prophase are the same as those described above for Hop1 (Fig 1C) and Spo76/Pds5 (Fig 3C–3E). However, while Rec8-GFP makes bright continuous lines at WT leptotene (Fig 4A, top), staining becomes progressively punctuate from late zygotene on, and at pachytene (Fig 4A, bottom) lines are never as bright and smooth as those of Hop1-GFP (Fig 1C) or Spo76-GFP (Fig 3A, left). Rec8 exhibits the same progressive localization pattern, with progressive changes, in absence of Hop1 (Figs 4B and S3F).

Further, Rec8-GFP and Spo76-Tred colocalize perfectly during leptotene (S3F Fig) and into mid-zygotene (Fig 4B, top). This perfect colocalization is, however, disrupted at pachytene where Spo76 remains only visible as stretches (Fig 4B, bottom). Retention of cohesin complexes in *hop1/asy1/hormad1* mutants is observed also in plants, *C*. *elegans* and mouse [10, 29–32]. The reason for the differential loss of Spo76/Pds5 *versus* its cohesin partner in *Sordaria*, remains unknown.

We found next that *rec8Δ* and *spo76-1* mutants (a non-null mutant of Spo76/Pds5, *PDS5* being an essential gene in *Sordaria*), exhibit analogous defects as *hop1Δ* after onset of synapsis. In WT, Hop1-GFP and Spo76-GFP exhibit 7 SC-correlated lines at pachytene (Fig 4C, left and 4D, left, respectively), whereas *rec8*Δ pachytene nuclei always exhibit a larger number of shorter lines of both Hop1-GFP (Fig 4C, right) and Spo76-GFP (Fig 4D, right). Analogously, in *spo76-1*, Hop1-GFP (Fig 4E) and Rec8-GFP (Fig 4F) also exhibit 13 to 25 such lines (*n* = 33 and 18 nuclei, respectively). Notably, while Hop1-GFP exhibits smooth staining in WT pachytene nuclei (Fig 4C), during mid-late pachytene, Hop1 shows a patchy staining in synapsed regions of *rec8Δ* (S3G Fig) and *spo76-1* (S3H Fig), suggesting that axis status is also abnormal even in regions where SC is present.

Comparisons among different mutant situations with respect to length and number of visible pachytene segments gives further information, as provided by two-tailed Student’s tests (Fig 4G and 4H). (i) The total length of the Hop1 and Spo76 segments in *rec8Δ* are significantly different (*P* < 0.01) while the Spo76 segments in *rec8Δ* and those of Hop1 in *spo76-1* are similar (*P* < 0.05; Fig 4G). (ii) The number of Hop1 and Spo76 segments in *rec8Δ* are not significantly different (*P* < 0.05; Fig 4H) while those of Hop1 lines in *spo76-1* are 1.6- to 1.7-fold higher (*P* < 0.0001; Fig 4H). This latter difference can be due to the fact that only short stretches of SCs/Sme4-GFP are formed in *spo76-1* (S4A Fig; *n* = 30 nuclei), likely corresponding to the thicker lines seen in Fig 4E, left, the rest of the lines marking un-synapsed stretches. Statistics could not be applied to the Rec8 lines in *spo76-1* because both their number (12 to 23) and their lengths (10 to 25 microns) are highly variable from one nucleus to the other (*n* = 25).

The observations presented show a functional interplay among Rec8, Spo76/Pds5 and Hop1 for maintenance of axis integrity at pachytene. Hop1 and Spo76 are mutually stabilizing: absence or defect of one component leads to partial depletion of the other. The same is true for Rec8 and Spo76. In contrast, Rec8/Hop1 interplay is unidirectional: absence of Rec8 results in depletion of Hop1, but absence of Hop1 does not result in depletion of Rec8 (discussion).

### Analysis of haploid meiosis shows that the SC and Spo76/Pds5 defects seen in *hop1Δ* are both dependent on Hop1 per se and not an indirect consequence of aberrant homolog pairing

Identification of synapsis and Spo76 localization defects along *hop1Δ* homologs during pachytene (above) raised the possibility that these defects might be a secondary consequence of a prior mutant defect in coalignment/pairing. To exclude this possibility, we examined prophase events during haploid meiosis where, in absence of a homolog, no homolog pairing occurs. We showed previously that a mutant lacking the Slp1 SUN-domain protein is defective for the nuclear fusion (“karyogamy”) that precedes meiotic prophase in *Sordaria*, but not for regular progression of meiotic events in the 2 unfused twin-haploid nuclei [33]. Specifically, in this mutant, the entire program of SC formation and recombination occurs efficiently between sister chromatids in each of the 2 nuclei. Also, just as in WT, in *slp1Δ*, Spo76-GFP loads as smooth lines along the 7 chromosomes (S4B Fig) and remains present in this condition throughout pachytene.

We now find that absence of Hop1 confers the same constellation of defects in these twin *slp1Δ* haploid nuclei as in diploid meiosis. (i) In the double *hop1Δ slp1Δ* mutant, Spo76-GFP forms continuous lines at leptotene by ascus size (S4C Fig) as in the *hop1Δ* single mutant (S4B Fig). However, at later stages, a mixture of short and longer Spo76 lines is present in all double *hop1Δ slp1Δ* nuclei (arrows in S4D Fig; *n* = 20 nuclei) just as in the diploid *hop1Δ* single mutant (Fig 4E), implying that the protein is being lost from some chromosome regions. (ii) In the single *slp1Δ* mutant, the SC central component Ecm11-GFP exhibits continuous seven lines in both nuclei with Hei10 foci in most of the lines (arrows in S4E Fig). In the double *hop1Δ slp1Δ* mutant, Ecm11-GFP (with Hei10 foci, arrows in S4F Fig) exhibits more numerous and shorter lines (S4F Fig, middle) than in the single *slp1Δ* mutant (S4E Fig), implying that Ecm11 and thus the SC is never complete along the seven chromosomes (*n* = 20 nuclei).

These results show clearly that defective SC formation in diploid *hop1Δ* and correlated loss of Spo76/Pds5 from axes which are not linked by an SC, cannot reflect aberrancies in the pairing process in the mutant because the *hop1Δ slp1Δ* double mutant shows similar defects between the sister chromatids.

### In absence of Hop1, recombination complexes still develop normally along chromosome axes in leptotene and in pachytene regions that acquire an SC

**Fig 5:**
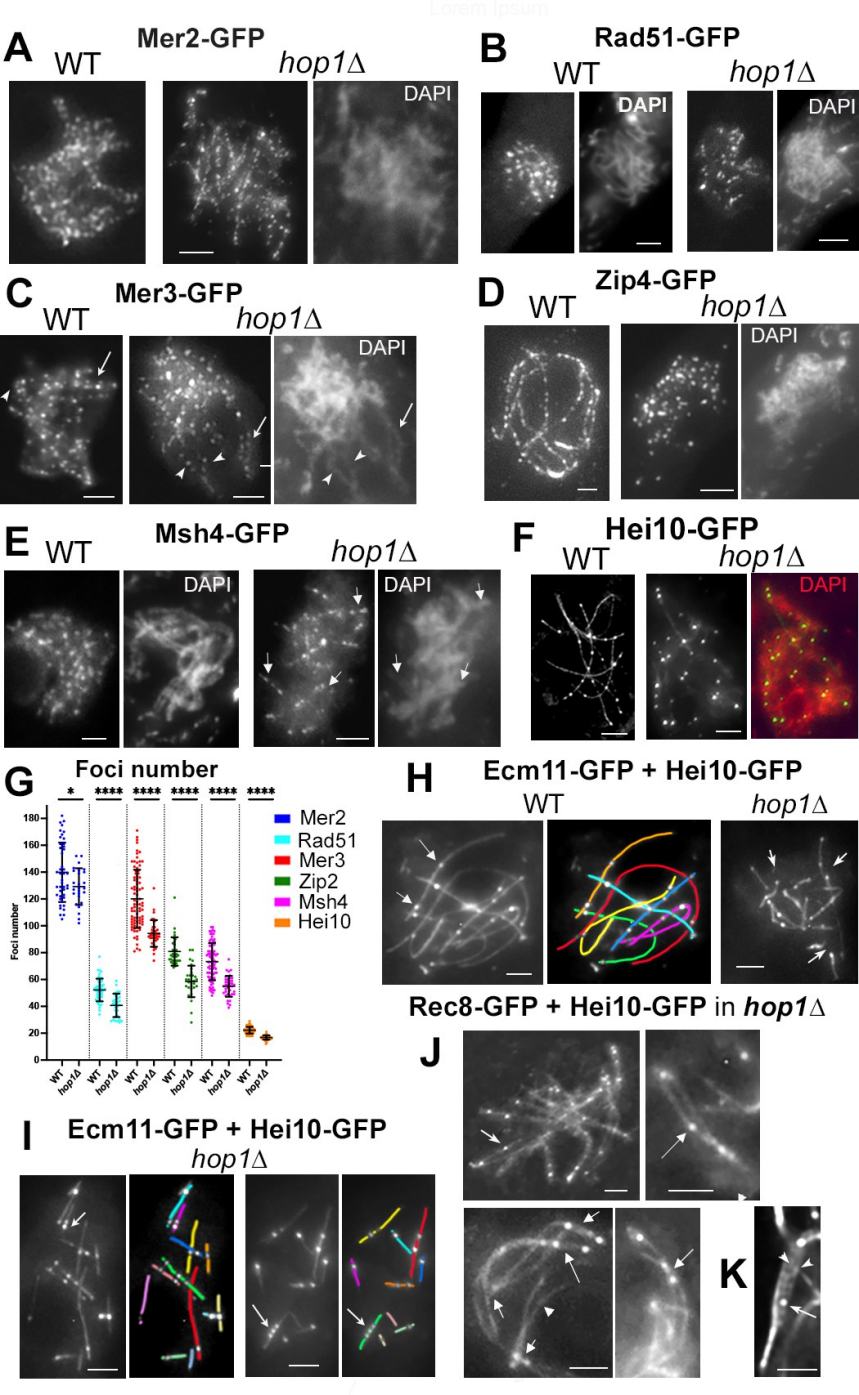
Localization of 6 recombination proteins in *hop1Δ*. (**A**) Mer2 forms regularly spaced foci along all leptotene chromosomes in WT (left) and in *hop1Δ* (right, with corresponding DAPI). (**B**) Rad51-GFP forms fewer foci likely because of rapid turnover in both WT (left) and mutant; corresponding DAPI, right. (**C**) During leptotene, Mer3-GFP makes evenly spaced foci along the chromosome axes in WT (arrow, left) and *hop1Δ* (right, with corresponding DAPI). Foci are seen in matched pairs in the segments where homologs are coaligned (arrowheads for one pair and arrow for the other, middle and right). (**D**) At pachytene, Zip4 occurs as regularly spaced foci along all homologs in WT (left) while in *hop1Δ*, lines are shorter (right and corresponding DAPI). See Zip4 and Zip2 foci at leptotene in S4G and 4H Figs. (**E**) While in WT (left), foci are seen along all bivalents, Msh4 forms only short segments of foci in *hop1Δ* (arrows, right and more examples in S4I Fig). Note also the difference in diffuseness of the chromatin between WT and *hop1Δ*. (**F**) Hei10 forms bright T3 foci in both WT (left) and mutant (middle and right: colocalization with DAPI (red)). (**G**) Quantification of the number of recombination foci in WT (left) and mutant (right) for each of the above proteins. From left to right, *n* = 44, 26, 49, 30, 92, 36, 32, 31, 76, 38, 112, and 54 nuclei, respectively. Mean and error bar (SD) are indicated for each set. Significance between WT and *hop1Δ* nuclei was determined by two-tailed Student’s *t* test: *P < 0.05; ****P < 0.0001. **(H)** In WT, Hei10 foci (arrows) are regularly spaced along the 7 SCs marked by Ecm11-GFP (corresponding drawing right). Mutant Hei10 foci (arrows) are also located along Ecm11 lines, but in contrast with WT, the SCs are never continuous from telomere to telomere. (**I**) Further illustration of the discontinuities of Ecm11 lines in *hop1*Δ. Note also that Hei10 foci are either evenly spaced or very close (arrows). (**J**) Colocalization of Rec8 and Hei10-GFP at pachytene in *hop1Δ*. Top: Arrows point to a bivalent exhibiting a Hei10 focus on only 1 homolog in the nucleus (left) and in the enlarged single bivalent (right). Bottom left and middle: examples of pachytene *hop1Δ* bivalents. Left: 2 bivalents with foci at both ends (arrows) and 1 bivalent in which one chromosome end is not synapsed (arrowhead). Right: bivalent with terminal and middle region synapsis (arrow). (**K**) Example of a Hei10 focus (arrow) located between 2 sister chromatids (arrowheads). Scale bars: 2 μm. The raw data underlying panel 5G are available in S1 Data. SC, synaptonemal complex; WT, wild-type.

Meiotic recombination and recombination-mediated coalignment occur through protein complexes that are directly associated with chromosome axes and, correspondingly, require components of that structure (e.g., [34,35]). Hop1 being an axis component, we assessed the role of *Sordaria* Hop1 in recombination complex/axis association, taking advantage of our large panoply of GFP-tagged versions of involved molecules. Analyses of 6 of the recombination proteins required for crossover formation (their foci number in *hop1Δ* versus WT is summarized below in Fig 5G) show clearly that Hop1 is not directly required for development of recombination complexes or for their association of with either axes or SCs.

Like in WT (Fig 5A, left), **Mer2**, required for axis-associated pre-DSB complexes and DSBs [25, 35] occurs as rows of regularly spaced foci along all chromosomes at leptotene in *hop1Δ* (Fig 5A, right), and in WT-like numbers (129.2 ± 13.6 versus 140 ± 22 in WT), confirmed by two-tailed Student’s *t* test of *P* < 0.05 (see Fig 5G; *n* = 26 and 44 nuclei, respectively). In the absence of Hop1, **Rad51** foci (Fig 5B, right), which mark DSB sites, show a statistically significant 22% reduction when compared to WT: 40 ± 8.7 versus 52.2 ± 8.5 in WT (two-tailed Student’s *t* test; *P* < 0.0001; *n* = 30 and 49 nuclei, respectively, Fig 5G). Thus, like *A*. *thaliana* ASY1 [10], *Sordaria* Hop1, has no strong role for DSB formation.

WT **Mer3** foci form initially on all chromosome axes at early leptotene (Fig 5C, left) and then, in coaligned regions, occur in pairs [36]. In absence of Hop1, Mer3 foci appear initially as evenly spaced foci along individual axes and are seen in matched pairs in the few regions where homolog axes are visibly coaligned (arrows in Fig 5C, right). The number of Mer3 foci is reduced by 21% when compared to WT: 94.3 ± 10 versus 120.1 ± 21.7 in WT (two-tailed Student’s *t* test *P* < 0.0001; *n* = 36 and 85 nuclei, respectively; Fig 5G). Hop1 is thus not required for Mer3 focus formation and axis association per se but is rather required for WT-like coalignment which, in turn, is required for development of matching pairs of Mer3 foci along all homologs.

**Zip2** and **Zip4** play a central role in nucleation and early extension of SC formation at zygotene [28,37]. Like in WT, foci of Zip2 and Zip4 are already present along chromosome axes at early leptotene in *hop1Δ* (S4G and S4H Fig, respectively) and again with a 27% reduction when compared to WT (58.5 ± 11.7 versus 80.9 ± 11, two-tailed Student’s *t* test *P* < 0.0001; *n* = 31 and 32 nuclei, respectively; Fig 5G). Thereafter, foci remain throughout pachytene, but contrary to WT where they are visible as regular rows of foci along all homologs (Fig 5D, left), in *hop1Δ*, they are seen as rows of foci that occur likely only in the regions that have formed SCs (Fig 5D, right).

**Msh4,** the meiosis-specific MutS homolog, stabilizes Holliday Junctions by encircling the involved duplexes (e.g. [38]). In WT *Sordaria*, Msh4 ensures full homolog juxtaposition, appears as foci on SC central region when SCs form and remains there in progressively decreasing numbers until mid-pachytene [36]. Contrary to WT where Msh4 forms continuous lines of foci along homologs (Fig 5E, left), in *hop1Δ*, Msh4 forms more or less long rows of foci (usually 11–15 rows in the 30 pachytene nuclei analyzed) which presumptively correspond to the 12–15 segments of SCs seen in the mutant (arrows in Fig 5E, right, and S4I Fig for more examples). The number of foci is correspondingly significantly reduced by 25%: 54.9 ± 7.8 compared to 73.3 ± 13.9 in WT (two-tailed Student’s *t* test *P* < 0.0001; *n* = 38 and 76 nuclei, respectively; Fig 5G).

**E3 ligase Hei10** forms bright foci at the sites of crossovers along WT mid-late pachytene SCs (Fig 5F, left; [39], and below). In *hop1Δ* similar foci are observed (Fig 5F, right) but their number is reduced by 24% when compared to WT (16.8 +/− 1.6 versus 22.2 +/− 2.5; two-tailed Student’s *t* test; *P* < 0.0001; *n* = 54 and 112 nuclei, respectively; Fig 5G and see below for detailed analysis).

Conclusion: Interestingly, there is a reduction of approximately 25% in the numbers of Rad51, Mer3, Msh4, and Hei10 foci in *hop1Δ* as compared to WT. This finding points to a reduction of approximately 22% in DSBs (by Rad51 and Mer3 foci) which is then propagated through later stages of recombination. Aside this rather modest effect, Hop1 is not, per se, required for normal development and structure association of recombination complexes throughout leptotene. And during pachytene, recombination complexes that normally exhibit association with the SC are again found in regions where SC is formed but are not found in regions where SC is absent, in accord with the aberrant status of these regions. We note also that the 24% decrease of Hei10 foci seen in *Sordaria hop1Δ* contrasts with more severe reductions of crossovers seen in budding yeast, mouse, *Brassica rapa*, rice, *C*. *elegans* and *A*. *thaliana* (e.g. [3,8,22]).

### In absence of Hop1, crossover localization is altered in synapsed regions with recombination interactions undergoing inter-sister repair in un-synapsed regions

As shown by colocalization with Ecm11-GFP as a SC marker, Hei10 foci occur all along the 7 homologs in WT (arrows in Fig 5H, left). In *hop1Δ*, foci occur also in association with the SC, but are restricted to the 11–17 Ecm11-GFP SC segments formed per nucleus (arrows in Fig 5H, right and 5I). Specifically, analysis of 552 of such SCs segments, (from 42 mid-pachytene nuclei) contain either no foci (15%), 1 (44%), 2 (33%), 3 (6%), or 4 (2%) Hei10 foci.

In WT, Hei10 foci are evenly spaced along the SCs (Fig 5F and 5H, left) as a consequence of the operation of crossover interference [39]. In *hop1Δ*, the average inter-focus distance is half that in WT (1.35 ± 0.93 versus 2.5 ± 1.12 microns in WT). This difference is reflected in 3 other features. First, in the mutant, the number of Hei10 foci is reduced by only 24%, whereas the total length of SC segments is reduced by approximately 40% the total WT length (above). Second, Hei10 foci are often seen closely spaced along SC segments (arrows in Fig 5I, left and right), which is not the case in WT (Fig 5F and 5H). Third, linear regression analysis between the number of Hei10 foci per nucleus and the SC lengths (in micron) per nucleus indicates that the CO number scales directly with axis/SC length like in WT (S5A Fig). However, the 2 slopes are significantly different, in accord with the fact that the average distance between the Hei10 foci is smaller in the mutant.

Crossover foci also exhibit an altered distribution along *hop1Δ* SC segments. The distribution of inter-crossover distances was fit to a gamma distribution, where the ν (shape) parameter reflects deviation from a Poisson distribution. Measurement of Hei10 foci in 552 *hop1Δ* SCs, compared to 658 SCs of WT, defines **ν** values of 2.77 ± 0.22 in the mutant compared to 5.03 ± 0.18 in WT. This reduction can be explained in several ways according to whether/how the crossover patterning has been perturbed by absence of Hop1 [40].

Furthermore, among the 146 *hop1Δ* bivalents traced in the pachytene nuclei marked by Rec8-GFP and Hei10-GFP (Figs 5J and S5B): (i) 25% exhibit a Hei10 focus on only 1 homolog in the non-synapsed regions (arrows in Fig 5J, top). Hei10 foci on single chromosomes has also been observed in haploid meiosis of *Sordaria*, budding yeast, *Drosophila melanogaster* and *A*. *thaliana* (e.g., [33,41]; reviewed in [34,42,43]) or on univalents in absence of *A*. *thaliana* ASY1 [44]. (ii) Approximately 73% show SC formation (with 1 to 3 Hei10 foci) at both ends (arrows in Fig 5J, bottom, left), 21% showed SCs (with 1 to 2 foci) only at 1 end, the un-synapsed other ends being more or less widely open (arrowhead in Fig 5J, bottom, left) and only 6% showed SC formation in the middle part of the bivalents, all containing at least 1 Hei10 focus (arrow in Fig 5J, bottom, right). Interestingly, recombination interactions undergo inter-sister repair in destabilized regions: on the unsynapsed chromosome containing a Hei10 focus (arrow in Fig 5K), the sister-chromatids are usually separated (arrowheads in Fig 5K). We infer that, at these sites, DSBs that were originally designated to be interhomolog crossovers are repaired instead by redirection to inter-sister recombination like in haploid meiosis (above and [33]).

Accordingly, there are no visible broken chromosomes at diplotene (S5C Fig left) and metaphase I in *hop1Δ* (*n* = 20 nuclei; S5C Fig middle left). Thus, all DSBs are repaired in one way or another, even in regions where synapsis is defective. However, 2 among the 10 metaphase I nuclei, showed early homolog separation (arrows in S5C Fig middle, right) and 1 among the 10 anaphase I nuclei exhibited 1 lagging chromosome (arrow in S5C Fig, right), which could explain the presence of abnormal ascospores in *hop1Δ* (S2B Fig).

### The Hop1 HORMA domain is critical for all Hop1 functions

**Fig 6:**
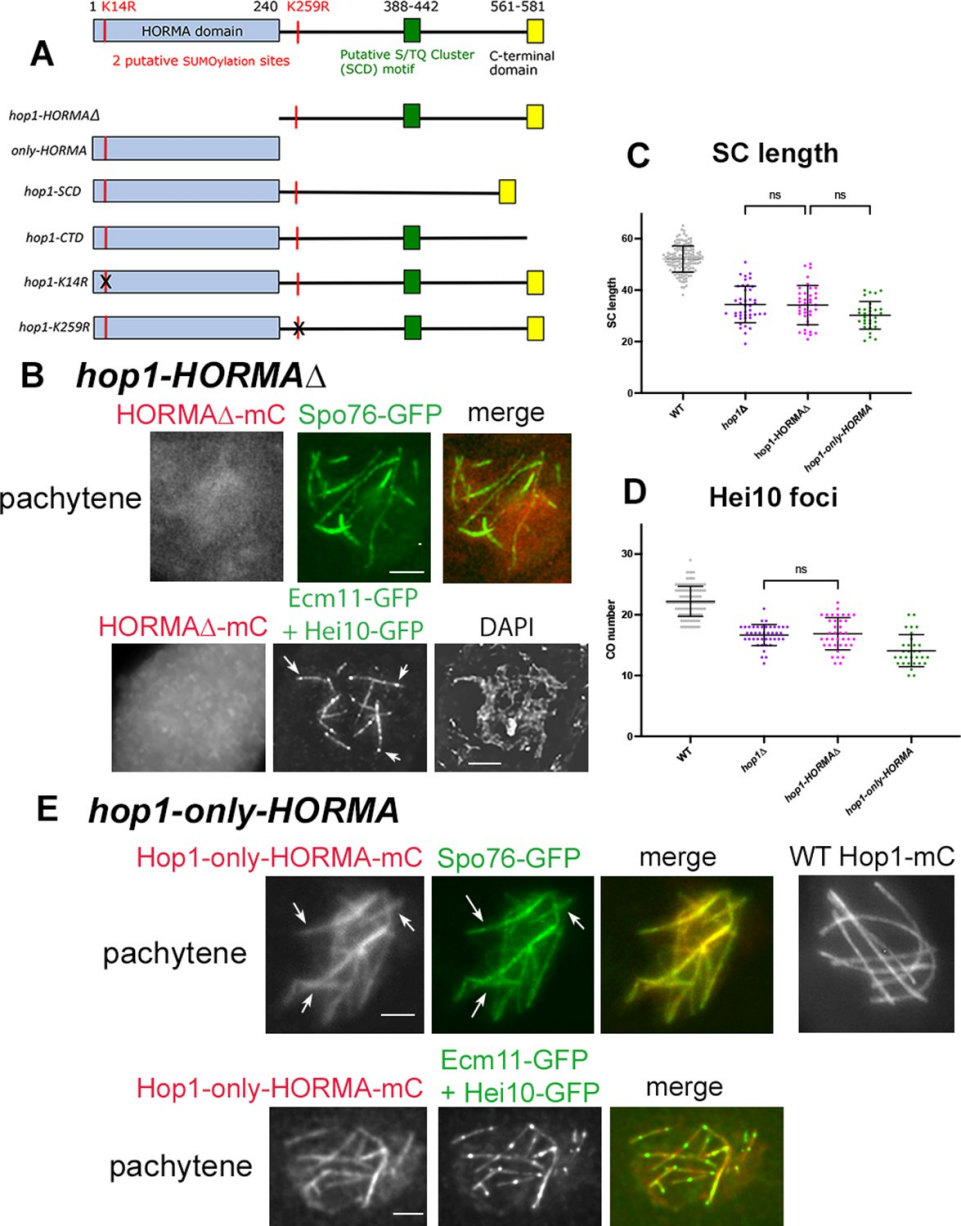
*Sordaria* Hop1 domain diagram of WT and mutants, and phenotypes of the 2 HORMA-domain mutants. (**A**) Top: domain diagram of WT Hop1. Bottom: diagram of the deleted or mutated Hop1 sites in the 6 analyzed mutants (). (**B**) Protein localization and phenotypes of the ***hop1-HORMAΔ*** mutant. Top: In the *hop1Δ* background, the mutant protein tagged with mCherry (mC, left) is not visible along the axes, marked by Spo76-GFP (middle) and merge (right). Bottom: colocalization with Ecm11-GFP + Hei10-GFP (middle) indicates that only few SC segments (containing Hei10 foci, arrows) are formed in this mutant; right corresponding DAPI. (**C, D**) *hop1-HORMAΔ* SCs exhibit the same length (**C**) and Hei10 foci number (**D**) as *hop1Δ*. Mean and error bar (SD) are indicated for each set. Significance between WT, *hop1Δ* and *hop1-HORMAΔ* nuclei was established by Brown–Forsythe ANOVA test: ns = not significant, *P*-value > 0.05; *n* = 38 and 42 nuclei. (**E**) ***hop1-only-HORMA*.** Top: the HORMA domain alone is sufficient for axis localization of the protein but only as discontinuous segments (arrows) that colocalize with Spo76-GFP (arrows middle and merge right) in contrast to the continuous lines seen in WT with Hop1-mCherry (right). Bottom: colocalization of Hop1-only-HORMA-mC (left) with Ecm11-GFP and Hei10-GFP (middle) and merge (right) indicates that the visible mutant segments correspond to SCs. (**C**) The mutant SCs have the same length as *hop1Δ* SCs (**C**) but exhibit a slightly lower number of Hei10 foci (**D**): Brown–Forsythe ANOVA test, ns = not significant; *n* = 31 and 42 nuclei. Scale bars: 2 μm. The raw data underlying panels 6C and 6D are available in S1 Data. SC, synaptonemal complex; WT, wild-type.

To further investigate the roles of Hop1, we generated and analyzed 6 mutant alleles that express partial or mutated forms of the Hop1 protein (Fig 6A). Each mutant gene was further tagged with mCherry at its C-terminus and introduced into the *hop1Δ* background for analysis. All of these alleles (as well as *hop1Δ*) allow ascospore formation but fail to efficiently form wild-type like 8-spored asci (S2B Fig). Also, prophase progression is impeded in absence of WT Hop1: all 7 analyzed mutants exhibit a 24-h prolongation of the period comprising coalignment and synapsis.

Among the mutants that retain a portion of Hop1, 2 were specifically designed to probe the role of the HORMA domain in the *hop1Δ* background: ***hop1-HORMAΔ***, in which the HORMA domain is deleted and ***hop1-only-HORMA*** in which only the HORMA domain is conserved (Fig 6A). As expected, the HORMA domain is critical for all Hop1 functions. When this domain is specifically deleted (in ***hop1-HORMAΔ***), the corresponding protein (tagged with mCherry) does not localize to chromosomes (Fig 6B, top, left) and the mutant exhibits a null phenotype in all respects. (i) At pachytene only pieces of Spo76-GFP (Fig 6B, top, middle) and Ecm11-GFP (Fig 6B, bottom) are visible. The SCs marked by Ecm11 contain; however, 1 to 3 Hei10 foci (arrows in Fig 6B, bottom, middle) like in *hop1Δ* (above). (ii) The mutant SCs show the same SC length and number than the *hop1Δ* SCs: 34.2 ± 7 microns and 12 ± 2 segments (*n* = 38 nuclei) versus 31 ± 9.1, and 15.6 ± 1.2 (*n* = 42 nuclei) confirmed by Brown–Forsythe ANOVA *P*-value > 0.05 (Fig 6C). They exhibit also the same number of crossover-correlated Hei10 foci (Fig 6D): 17 ± 3 (*n* = 38) as the null *hop1Δ* mutant (16.8 ± 1.6; *P* > 09; *n* = 54 nuclei).

Analysis of the complementary allele comprising the HORMA domain alone (***hop1-only-HORMA***) reveals the interesting fact that the HORMA domain alone is sufficient for chromosome localization from leptotene (S6A Fig) through pachytene (Fig 6E). However, even at leptotene, the “only-HORMA” protein displays a more discontinuous signal than WT Hop1 (S6A Fig left versus right). And, at pachytene, the mutant protein is only visible as more or less long segments that colocalize both with Spo76-GFP (arrows in Fig 6E, top, left) in contrast with the WT Hop1-mCherry localization along the 7 homologs (Fig 6E, right). Colocalization with Ecm11-GFP (Fig 6E, bottom) shows that the segments that exhibit HORMA-only-mC correspond to SC stretches. Their number and lengths: 11 ± 2 and 30.2 ± 5.4 microns (*n* = 31 nuclei) are again almost the same as in *hop1Δ*: 15.6 ± 1.9 and 31.7 ± 9.1 microns (*P* > 09; *n* = 54 nuclei) (Fig 6C). Together, these findings show that the *hop1-only-HORMA* mutant has essentially a null phenotype for Hop1 function and that this phenotype also involves a tendency for destabilization of Hop1 binding itself. Additionally, the number of crossover-correlated Hei10 foci of 14 ± 2 (*n* = 31 nuclei) is significantly lower than in *hop1Δ* and *hop1-HORMAΔ* (*P* < 0.001; Fig 6D), suggesting that HORMA-domain localization actively interferes with Hei10-mediated recombination in an allele-specific “dominant-negative” effect.

### The C-terminal domain of *Sordaria* Hop1 is not required for Hop1 loading but is crucial for both pairing and chromosome-entanglement resolution, despite absence of a clear “closure motif”

**Fig 7:**
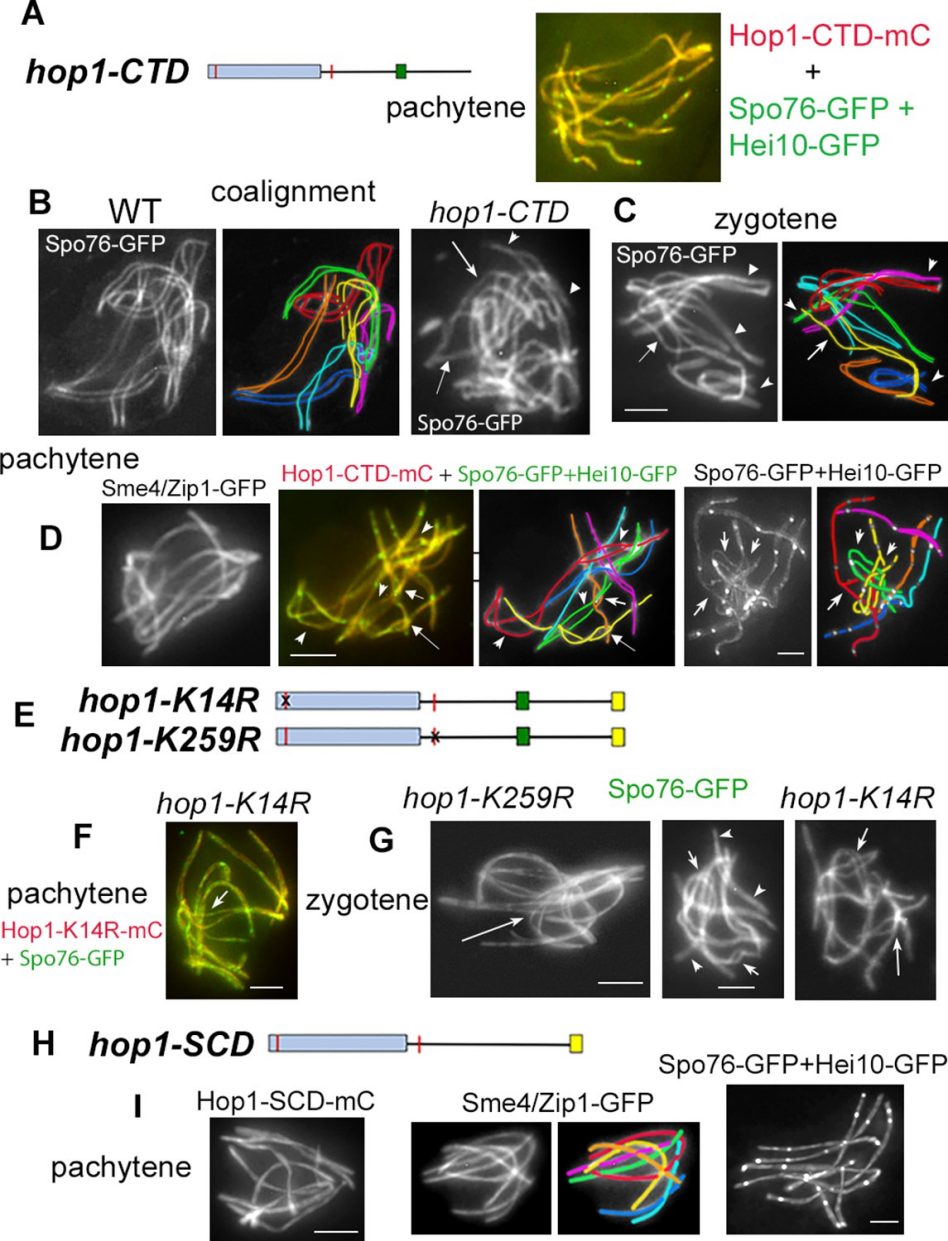
Coalignment, SC and CO/Hei10 phenotypes of the 4 non-null *hop1* mutants. (**A–D**) ***hop1-CTD*** mutant. (**A**) Pachytene nucleus illustrating the perfect colocalization of Hop1-CTD-mC with Spo76-GFP (“green” Hei10 foci are visible along all bivalents). (**B**) Late leptotene coalignment stage (axes marked by Spo76-GFP). The 7 WT homologs are synchronously coaligned (left and middle) while the *hop1-CTD* mutant shows highly irregular coalignment with some ends synapsed (arrowheads) when others (arrows) are still largely separated. (**C**) Irregularities persist into zygotene: most ends are synapsed (arrowheads) but one homolog end (arrow and yellow in drawing) is still widely open. (**D**) Three pachytene nuclei. Left: example of a nucleus with full synapsis (indicated by Sme4/Zip1-GFP). Middle and right: mid- and late pachytene nuclei (and corresponding drawings) with large open regions (arrowheads in middle nucleus) and interlockings (arrows in both). (**E–G**) ***hop1-K14R*** and ***hop1-K259R*** mutants. (**E**) Sites of point mutations are indicated by a black cross on red lines. (**F**) Pachytene nucleus with perfect colocalization of Hop1-K14R-mC with Spo76-GFP. Arrow indicates the site where 2 bivalents are trapped in the non-synapsed region of a third one. (**G**) Three examples of delayed/abnormal coalignment/synapsis. Arrows point to interlocking sites (left and right nuclei) and arrowheads indicate synapsed regions in the middle nucleus. (**H, I**) ***hop1-CTD*** mutant. (**I**) At pachytene, the mutant protein localizes along all axes (left nucleus). The mutant exhibits also regular synapsis as shown by Sme4-GFP (middle nucleus and corresponding drawing) and regularly spaced Hei10 foci (right nucleus). Scale bars: 2 μm. CTD, C-terminal domain; SC, synaptonemal complex; WT, wild-type.

By sequence comparison, the last 63aa of *Sordaria* Hop1 correspond to both the type of charged and hydrophobic amino acids found in other organisms and the expected length of a “closure motif,” found at Hop1 C-termini in many organisms [24]. We created a ***Hop1-CTD*** mutant protein, with these 63 aa removed, and tagged it by mCherry. Hop1-CTD-mCherry colocalizes continuously with Spo76/Pds5-GFP along chromosome axes from leptotene throughout pachytene (Fig 7A) just as does Hop1 in WT (Fig 1C). The deleted domain is thus not required either to anchor the protein to the chromosome axis or for maintenance of normal axis integrity during pachytene. This phenotype is particularly notable because in some organisms, the C-terminal “closure motif” is known to mediate Hop1 axis localization (e.g., [2,3,6]). Interestingly, however, *A*. *thaliana* homolog ASY1 has a canonical C-terminal CM but does also not require this motif for axis association of the protein [23]. Thus, for both *Sordaria* and *A*. *thaliana*, the mechanism by which Hop1 becomes incorporated into axes, without benefit of a closure motif, remains to be defined. One possibility could be a direct interaction of Hop1 with cohesins. Such interactions are known in several organisms but involvement of the closure motif per se, remains unknown (e.g., [2]).

Using the advantage of continuous axis localization of Hop1-CTD-mC and its perfect colocalization with Spo76-GFP, we next analyzed in detail the pairing and synapsis phenotypes of the *hop1-CTD* mutant. During WT leptotene, the 7 homologs synchronously co-align first at 400 nm and then at 200 nm separation distance (Fig 7B, left) before proceeding to synapsis. In *hop1-CTD*, coalignment is severely defective, with some homologs still largely separated (arrows in Fig 7B, right) and others partially paired or synapsed (arrowheads in Fig 7B, right). In accord with these configurations, the mutant exhibits also more or less dramatic per-chromosome and per-region defects at zygotene with most ends synapsed but also un-synapsed ends (respectively arrowheads and arrow in Fig 7C).

Moreover, even at pachytene, SC formation (tested by Sme4/Zip1-GFP) is complete from telomere to telomere along the 7 homologs in only 15% of the 81 analyzed pachytene nuclei (Fig 7D, left). The other 85% nuclei show 1 to 5 non-synapsed regions per nucleus (arrowheads in Fig 7D, middle and in corresponding drawing; more examples in S6B Fig). All bivalents are affected but the 3 longer chromosomes are more often partially non-synapsed (in 50% to 40% of the nuclei) than the smaller chromosomes (30% to 18%). The presence of fully synapsed chromosomes, as well as the continuous localization of Spo76-GFP along the axes (above), implies that non-synapsed regions in this mutant are due to defects in homologous pairing and not to abnormal axis status as seen in *hop1Δ*.

Interestingly, the mutant most impressive defect is the presence of a high number of topological irregularities (“interlocks/entanglements”) visible throughout pachytene (arrows in Figs 7D, middle and right, and S5B). Chromosome entanglements do also occur in 20% of WT zygotene nuclei (*n* = 50 nuclei), but they are absent at pachytene (*n* = 120), implying active resolution [36]. In contrast, interlock configurations are still present in 41% (33/80) of *hop1-CTD* late pachytene nuclei (Figs 7D, right and S5B, right), implying a specific defect in interlock resolution. Due to weak Rec8 pachytene “staining” and absence of Spo76 in non-synapsed regions (above), we were not able to count the number of interlockings in *hop1Δ*, but the fact that all analyzed zygotene nuclei showed at least 1 entanglement (Fig 3C and 3D), could indicate that this defect is also present in the null mutant.

Moreover, we showed previously that in absence of Mlh1, 45% of the late pachytene nuclei exhibited at least 1 bivalent with an open region and/or an interlocking, implicating a role of Mlh1 in interlock resolution [36]. To test the role of Mlh1 in the *hop1-CTD* mutant background, we analyzed synapsis in the *hop1-CTD mlh1Δ* double mutant (S6C Fig). It exhibits even a greater number of interlocks than each of the single mutants at mid-late pachytene: 80% in *hop1-CTD mlh1Δ* (*n* = 25 nuclei) compared to 41% in *hop1-CTD* (*n* = 80), 45% in *mlh1Δ* (*n* = 300), and 0% in wild type (*n* = 120). Since regular homolog coalignment is required to avoid interlock formation, the role of Hop1 in pairing could explain this synergy. However, Mlh1 being present in the *hop1-CTD* mutant, there must be other factors/effects that impede removal of interlocks in the mutant and thus also likely in WT.

### The 2 putative SUMO sites are also required for efficient pairing and interlocking resolution but the conserved S/TJQ cluster domain (SCD) is not

We also mutated two of the identified putative SUMO sites creating point-mutations in the ***hop1-K14R*** and ***hop1-K259R*** alleles (red lines in Figs 6A and 7E). Axis integrity is normal: both mutant proteins (tagged by mCherry) and Spo76/Pds5-GFP colocalize continuously along chromosome axes from leptotene throughout pachytene in a *hop1Δ* background (Figs 7F and S6D) just as in WT (Fig 1C).

Synchronous coalignment is, however, defective, with some homologs still largely separated and others partially synapsed (arrows and arrowheads in Fig 7G, middle and right). In accord with these defects, both mutants exhibit complete SC formation only in, respectively, 44% (*n* = 50 nuclei) and 65% (*n* = 89) of the analyzed pachytene nuclei and again, the 2 longer chromosomes are more often un-synapsed than the 4 smaller chromosomes (e.g., 82% and 64% in *hop1-K14R*). Also, as in *hop1-CTD* nuclei, interlock configurations are visible at zygotene (arrow in Fig 7G, left) and persist throughout late pachytene (arrow in Fig 7F) in 38% (19/50) of the *hop1-K14R* nuclei and in 13% (12/89) of the *hop1-K259R* nuclei.

In contrast, the ***hop1-SCD*** mutant, in which the putative Zn-finger motif (comprising 54 aa) is deleted from the *HOP1* gene (Figs 6A and 7H), exhibits none of the chromosome defects characteristic of the null mutant phenotypes: (i) Hop1-SCD-mC is continuous along all axes from leptotene to pachytene (Fig 7I, left), where it colocalizes with Spo76-GFP (S6E Fig); (ii) 96% of nuclei show fully regular SCs (by Sme4/Zip1-GFP, Fig 7I, middle; *n* = 50 nuclei) implying that, both pairing and interlock resolution are WT-like and that the SCD motif is not required for these features. (iii) Hei10 foci form along all 7 bivalents (Fig 7I, right) like in WT, but further analyses show defects in their localization (e.g., closely spaced foci) and number, indication of a role of this domain in the crossover patterning (below).

Unexpectedly, when compared to WT, this mutant still produces a reduced number of 8-spored asci in the *hop1Δ* background (S2B Fig). As we observed no obvious precocious homolog separation in the 20 metaphase I nuclei of the mutant, we have no clear explanation for this phenotype. It was shown that in budding yeast, the pericentromeric regions are under-enriched for Hop1 [45]. We do not know the status of Hop1 at the *Sordaria* centromeres, but if the same pattern is true, we cannot exclude an alteration of chromatid cohesion in the mutant.

### All 4 “non-null” mutants exhibit increased axis length

In the 2 *hop1* point mutants, as well as in the *hop1-SCD* and *hop1-CTD* mutants (hereafter referred to collectively as the 4 “non-null” mutants), axes assemble correctly with WT-like localization of Spo76-GFP and Rec8-GFP. Moreover, SC forms along the lengths of all homologs. However, we also discovered that all 4 mutants exhibit 25% longer axes/SCs than WT (Fig 7A): 65.1 ± 5.7 microns in ***hop1-SCD*** (*n* = 54), 69.2 ± in ***hop1-CTD*** (*n* = 49), 65.1 ± 4.1 in ***hop1-K14R*** (*n* = 53 nuclei), and 64.5 ± 6.5 in ***hop1-K259R*** (*n* = 45), when compared to the 52.2 ± 5.1 microns in WT (*n* = 146). Interestingly, while significantly different from WT (Brown–Forsythe ANOVA *P* < 0.0001), the observed increases are not significantly different among the 4 mutants (*P* > 0.05). Furthermore, mutant axis lengths are already longer at the coalignment stage, thus before SC formation, indication of an early role of Hop1. Axes lengths at late zygotene and early pachytene in *hop1Δ* (by Rec8-GFP) are also longer than in WT: 70.7 ± 3.8 microns (only 10 nuclei measured, due to the weak Rec8 signal). These results reveal that Hop1 has a discrete function that impacts axis length during axis assembly.

### Non-null mutant phenotypes reveal a direct role for Hop1 in crossover interference

**Fig 8:**
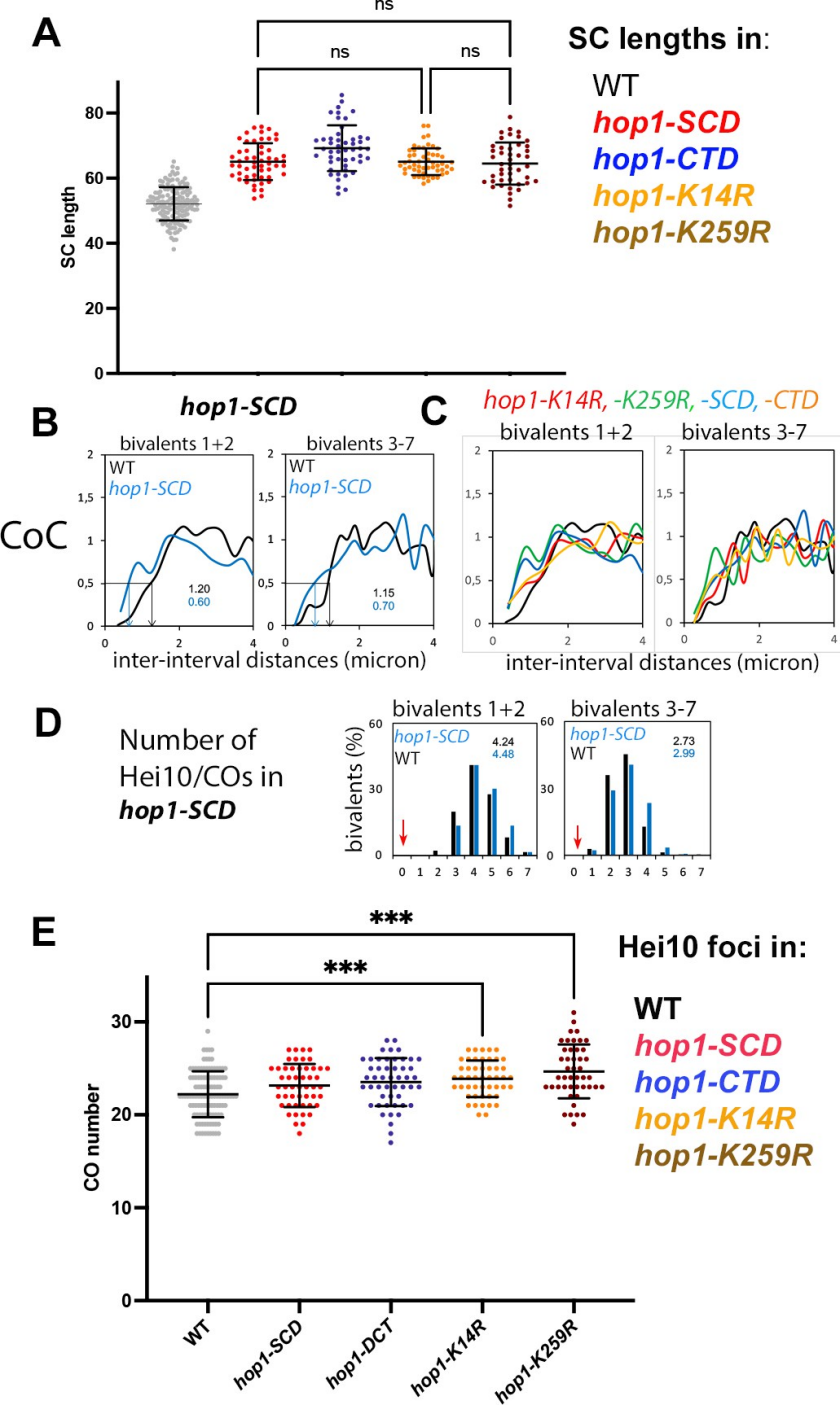
SC lengths, crossover number, and distribution in the 4 non-null mutants. (**A**) Comparison of the SC length per nucleus (in microns) of the 4 non-null mutants vs. WT SC lengths, indicates that their length is different from WT (gray, left) but not significantly different among the 4 mutants (Brown–Forsythe ANOVA *P* > 0.05; ns = not significant). Mean and error bar (SD) are indicated for each set. (**B, C**) CoC relationships for positions of correlated Hei10 foci/crossovers along (**B**) *hop1-SCD* and (**C**) *hop1-K14R*, *hop1-K259R* and *hop1-CTD* pachytene bivalents (longer 1 and 2 bivalents left, 3 to 7 bivalents right). (**B**) The strength of crossover interference is provided by the inter-interval distance at which CoC = 0.5. This “interference distance” (see details in Methods) is 1.30 microns in WT (black) and 0.6 microns in *hop1-SCD* (blue). These values correspond approximately to the average distance between adjacent crossovers. (**C**) The same difference between WT (black) and mutants (red, green, and orange lines) is seen in the CoC curves of the corresponding mutants. CoC curves indicate that the 4 mutants show interfering COs but with smaller than WT interference distances. (**D**) Number and distribution of Hei10 foci along the *hop1-SCD* SCs of the 2 long bivalents (bivalents 1 and 2; left) and the other shorter bivalents (bivalents 3–7; right); red arrows indicate the absence of E0 bivalents, which means that all bivalents exhibit at least 1 Hei10 focus. (**E**) Comparison of the number of Hei10 foci per nucleus in the 4 non-null mutants vs. WT (gray). The number of foci is not significantly different among the 4 mutants. Only the 2 SUMO-site mutants (right) show slightly higher numbers of foci than WT (ANOVA *P* < 0.0004; see text for the number of nuclei analyzed). Mean and error bar (SD) are indicated for each set. The raw data underlying all panels from 8A to 8E are available in S1 Data. CoC, coefficient of coincidence; SC, synaptonemal complex; WT, wild-type.

In the ***hop1-SCD*** mutant, where SC formation is highly regular (above Fig 7I), crossover interference can be examined without complexities due to incomplete synapsis. In this mutant, crossover interference is defective by 2 criteria. First, analysis of crossover interference by coefficient of coincidence (CoC) reveals a substantial increase in the probability of closely spaced double crossovers in both long and short bivalents (Fig 8B). Second, the gamma distribution of distances between adjacent Hei10 foci for 350 *hop1-SCD* bivalents exhibits a **ν** value of 2.71 ± 0.46 as compared to the 5.03 ± 0.18 seen for foci along 658 SCs of WT, consistent with defective interference.

Furthermore, the same effect is observed in the ***hop1-K14R*, *hop1-K259R*,** and ***hop1-CTD*** mutants, even though pairing/synapsis is often compromised (above): their gamma **ν** values are respectively of 2.7 ± 0.44, 2.53 ± 0.41, and 2.69 ± 0.36 (versus 5.03 ± 0.18 in WT) and CoC analysis reveals, a substantial increase in double crossovers that are closely spaced in both long and short chromosomes (Fig 8C). Moreover, CoC curves show that crossover interference is similarly reduced in the ***hop1-K259R*** mutant, in nuclei with full SCs and nuclei with open regions in their SCs (due mostly to non-resolved interlockings; S6F Fig). On the other hand, there is no increase in the frequency of bivalents that fail to exhibit even 1 Hei10 focus, even in the smaller bivalents (Fig 8D, red arrows), indicating that occurrence of the so-called “obligatory crossover” remains intact, in contrast with the phenotype of the *A*. *thaliana asy1* mutant [46].

We note that the effects observed in the non-null mutants reflect likely a loss of normal Hop1 function rather than a gain of interfering function by the mutant proteins. This interpretation is supported by the fact that all 4 non-null mutants exhibit the same basic defects, many of which are also mirrored in the null mutant. Interestingly, two of the mutants (*hop1-K14R* and *hop1-K259R*) carry point mutations in two of the identified putative Hop1 SUMOylation sites, suggesting that SUMOylation of Hop1 is required for WT-like axis length and crossover patterns.

### The non-null mutants show WT crossover levels despite increased axis length

A general feature of meiosis is that, for a given organism, the density of crossovers per micron axis length remains constant across diverse situations (review in [47,48]). For example, a variation in axis length is accompanied by a proportional variation in the number of crossovers in human and *A*. *thaliana* male versus female (e.g., [44,47,48]) or in mutant situations in *Sordaria* (e.g., the 4 RNAi-related dicer and argonaute mutants [49]). However, intriguingly, the *hop1-CTD*, *hop1-SCD*, *hop1-K14R*, and *hop1-K259R* mutants exhibit essentially the same average total number of crossovers per nucleus as the WT strain (Fig 8E), despite the fact that all 4 mutants exhibit 25% longer axes (Fig 8A) and variable pairing/synapsis defects. Specifically, relative to WT, the number of crossover-correlated Hei10 foci per nucleus is not significantly increased in ***hop1-SCD*** (23.2 ± 2.3; *n* = 50 nuclei) or ***hop1-CTD*** (23.5 ± 2.6; *n* = 49) and only slightly increased (ANOVA *P* < 0.0004) in ***hop1-K14R*** (23.9 ± 2; *n* = 53) or ***hop1-K259R*** (24.7 ± 2.9; *n* = 45) when compared to 22.2 ± 2.5 Hei10 foci in WT (*n* = 112 nuclei). Also, as for SC/axis lengths (above), the number of Hei10 foci is not significantly different among the 4 mutants (ANOVA *P* > 0.05). Further, the reduction in the average crossovers per axis length cannot be attributed to incomplete synapsis of some bivalents because it is observed in ***hop1-SCD*** with full SC formation (above) and in the 15% of ***hop1-CTD*** nuclei where bivalents exhibit complete synapsis (SC length of 70.1 ± 7.2 microns and 23.6 ± 0.5 foci compared to 69.2 ± 7 and 23.5 ± 2.6 in the nuclei with defective pairing). Thus: all 4 *hop1* non-null mutants tend to maintain a constant number of Hei10 foci in situations where it might be expected that the number of crossovers would be increased (discussion).

## Discussion

The current study was initiated to further explore the roles of HORMA-domain protein Hop1 taking advantage of *S*. *macrospora* as an experimental system for analysis of meiotic chromosomal events. Phenotypic analysis of null and non-null mutants has revealed several new roles. We provide evidence that Hop1 is directly required for chromosome entanglement resolution, modulation of chromosome axis length, crossover patterning and interference. Most importantly, we identify a role for Hop1 and cohesin-related proteins in achieving a WT-like pachytene chromosome structure. Interestingly, despite these diverse roles, a reduction of only 25% in the number of recombination foci indicates that Hop1 is not required for recombination initiation or for progression of recombination to crossovers, with corresponding complexes forming normal axis/SC association.

### Hop1, cohesin Rec8 and cohesin-associated Spo76/Pds5 are required to maintain the chromosome structure at the transition from coalignement to SC formation

In wild type, leptotene coalignment brings homolog axes to approximately 200 nm separation, after which SC formation occurs. In the absence of Hop1, coalignment occurs. However, during the transition beyond this stage, axis-component Spo76/Pds5, which was previously localized continuously along the chromosomes, is no longer maintained in the regions that lack an SC. The *hop1-HORMAD only* mutant phenotype suggests that Hop1 itself also tends to be lost from these regions. Regions at the ends of chromosomes appear to be relatively insensitive to these effects.

As a result of these phenotypes, recombination complexes that would normally be present along pachytene SCs (Mer3, Msh4, Zip2/4, and Hei10) are absent from the regions lacking an SC. The only exception is that a few Hei10 foci still occur in un-synapsed regions, localized only to one of the 2 homologs, and often in a region with split sister chromatids, likely mediating inter-sister repair. Control experiments in haploid meiosis show that the observed axis defects are not an indirect consequence of aberrant homolog pairing in these regions.

Interestingly, similar internal regions defects occur also in *A*. *thaliana* in absence of Hop1 homolog ASY1: the *asy1null* mutant shows limited SC formation specifically in sub-telomeric regions, with accompanying development of crossover recombination complexes specifically in those regions, at increased density [44, 46]. Moreover, the *rec8 null* mutant of *A*. *thaliana* shows also severe defects in axis continuity and SC formation [44,50].

We found, furthermore, that analogous effects occur in the absence of Rec8 and in *spo76-1*, a non-null mutant of cohesin-associated *SPO76/PDS5*. Thus, Hop1, Rec8 and Spo76/Pds5 collaborate to ensure a regular progression from a leptotene configuration to a WT-like pachytene configuration. Collaboration among these molecules is not unexpected in light of many observations which document their physical and/or functional interaction and/or their close spatial proximity within chromosome axes (e.g., [1–5,7,20]).

Pachytene depletion of Hop1 and orthologs occurs throughout the chromosomes as a regular feature of the transition from leptotene to pachytene in budding yeast, mouse, and plants. In those organisms, Hop1 is abundant on axis at leptotene but is more or less depleted from axes concomitant with SC formation (references above) in a PCH2/TRIP13-dependent manner and dependent on SC components SYCP1 or TEX12; conversely, in absence of Pch2, or SYCP1 or TEX12, Hop1 remains on synapsed chromosomes [6,12,17,23,27]. This dynamic behavior allows perhaps to first promote the critical transition to SC formation and then to allow efficient removal of Hop1 by Pch2 at pachytene exit. It is interesting to speculate that the programmed depletion of Hop1 and orthologs in these organisms might somehow be related to the dramatic depletion of axis components that we observe in *Sordaria* in axis-compromised mutants.

### The critical Hop1/Rec8/Spo76-dependent event may be emergence of inter-axis DNA/structure bridges

The above findings raise one important fundamental question: Why is Hop1 specifically and dramatically required in some chromosomal regions when the chromosomes transit from the coalignment to the SC state? An attractive possibility is presented by the fact that this progression is mediated by a critical process in which the invisible recombination-related DNA connections that initially link coaligned axes (related to DSBs repair) are converted to robust bridges that, in turn, link the coaligned homolog axes ([28] and references therein). These bridges further mediate the relocation of recombination complexes from on-axis to between-axis positions and, by progressive shortening, nucleate SC formation [28].

We have not been able to examine bridge formation by high-resolution microscopy in *hop1* mutants for technical reasons, but several lines of evidence strongly suggest that the chromosome modifications observed in absence of Hop1 occur at this specific stage. (i) Spo76/Pds5 localization is unaltered during homolog coalignment prior to bridge formation or if chromosomes are arrested at late leptotene, immediately prior to onset of bridge emergence, due to absence of Sme4/Zip1. (ii) Bridges contain axis components as defined in several organisms: Spo76/Pds5 in *Sordaria* [28], the Hop1 homolog ASY1 in *Brassica oleracea* [15], and RAD21L in mouse [51], which, moreover is known to interact with HORMAD1 [52]. How bridges evolve is unknown, but axis modulation could be involved. (iii) In absence of Hop1 when some regions of a bivalent exhibit SCs and other regions are still at the coalignment stage, Spo76 is not yet lost from these non-synapsed regions (Fig 3C and 3D). This finding suggests that the observed effects are not a general consequence of global progression from leptotene to pachytene but, instead, are direct consequences of specific local chromosomal events. Also, asynchrony of this transition is characteristic of WT meiosis as seen in the appearance and disappearance of bridges along homologs, with SCs formed in some regions while others still show bridges [28].

We further note that bridge formation all along the axes might compromise their continuity and thus involve a tendency for “destabilization” of the axis structure per se. Moreover, the first discernible event of bridge formation is release of recombination proteins like Msh4 and Zip2/4 from axes to a “hanging” off-axis position [28], an event that could easily also lead to changes in the axis components/continuity. We therefore suggest that the role of the Hop1/Rec8/Spo76-Pds5 ensemble is to constrain axis reorganization, thereby channeling chromosome development into the appropriate outcomes. An analogous effect could explain the depletion of Hop1 from chromosome axes in WT meiosis at this stage in other organisms (above).

### Hop1 plays a direct role in homolog coalignment and interlock resolution

Hop1 is required for normal homolog pairing in *Sordaria*, as in mouse, plants and budding yeast (review in [2,3,8]). The current study shows that this requirement reflects direct participation of Hop1 in the pairing process and, moreover, that Hop1 has an additional role specifically for resolution of interlocks.

In most other studied organisms, notably mouse and budding yeast (but not *A*. *thaliana*; above), Hop1 is required for DSB formation, which is a key prerequisite for the pairing process, leaving open the question of whether Hop1 is required directly or only indirectly via its role for DSBs. Since even the *Sordaria hop1Δ* mutant forms approximately 78% of DSBs and recombination proteins including Mer3 (above), Hop1 is probably not required for making initial DSB/partner interactions in this organism, but is important for the ensuing juxtaposition of homolog axes in space (and likely correct bridge formation) in the homolog middle regions. As reduced axis stiffness was predicted to compromise spatial juxtaposition [36], a speculative possibility is that Hop1 is required for normal axis stiffness, the mechanism of which remains unknown. Our previous studies have directly implicated Mer3, Msh4, Zip2, and Zip4 as important players in coalignment with respect to both progression of axis juxtaposition and achievement of a topologically regular outcome [28,36]. We can now add Hop1 as a new player in these processes.

Analysis of the 4 *hop1* non-null mutants indicates also that Hop1 is important for minimizing and/or resolving chromosome entanglements. Inefficient axis juxtaposition might tend to create more interlocks, but if interlock resolution were efficient like in WT where all interlocks are resolved at early pachytene, none would remain by late pachytene. The fact that this is not the case in the 4 non-null mutants, in which SC formation is wild-type like, implies an additional Hop1 role specific to that process. It implies also a distinct role for interlock resolution independent of spatial juxtaposition of homolog axes.

We showed previously that Mlh1 plays a role in interlock resolution [36]. The same is true in the non-null *hop1* mutant background. This role could reflect Mlh1-mediated dissociation of stalled recombination intermediates and/or absence of Mlh1-mediated resolution of crossover-fated double Holliday junctions [36,47,53]. Interestingly, the *hop1-CTD mlh1Δ* double mutant exhibits even a greater number of interlocks than either single mutant. Perhaps an increase in interlocks simply numerically overwhelms Mlh1-mediated resolution capacity.

### Hop1 is important for spreading of the crossover interference signal and for maintenance of constant total crossover number despite axis length increase

Analysis of Hei10 focus positions (by CoC and gamma distribution analysis) along fully synapsed chromosomes in the non-null *hop1* mutants shows unambiguously that Hop1 is important for crossover interference along prophase chromosomes. Furthermore, there is no defect in ensuring occurrence of the obligatory crossover. Gamma distribution analysis suggests that crossover interference is also reduced in the *hop1 null* mutant. A reduction in even spacing as defined by gamma distribution analysis could result from either (or both) of 2 effects: an increase in the intrinsic probability that a particular precursor recombination interaction will undergo crossover designation and/or a decrease in the distance over which the inhibitory interference signal spreads [40]. Aberrant crossover patterning and interference is also observed in *A*. *thaliana asy1* mutants where recombination is largely restricted to chromosome ends [44,46]. The very strong suggestion, then, is that Hop1 is directly important for spreading of the interference signal along the chromosomes in both organisms.

The *hop1* mutant phenotypes point also to the existence of effects that tend to keep total crossover levels constant. First, in the SC stretches that form in the absence of Hop1, the density of crossovers is higher than in WT: the number of crossover-correlated Hei10 foci is reduced by only 20% as compared to WT, thus to a much lesser extent than total SC length (which is 60% the WT length). Such an effect has been described previously in mutants where SC formation is limited due to pairing defects, e.g. non-null *mer2 Sordaria* mutants and *A*. *thaliana* neddylation and kinesin mutants [25,54,55]. In the 3 cases, it seems that when the number of precursor interactions is reduced, crossovers form at elevated levels in regions that still retain such precursors.

Second, a role of Hop1 in keeping total crossover levels constant is also shown by the *hop1* non-null mutant phenotypes (above). The 4 mutants show essentially the same number of Hei10 foci (and thus crossovers) as WT meiosis. This is paradoxical because: (i) chromosome axes are 25% longer than in wild type, which normally is expected to result in a commensurate increase in total crossover number [review in [48]; and (ii) crossover interference is defective which, because it reduces the constraint that events must be evenly spaced, should also be associated with an increase in the number of crossovers [40]. This could mean that when Hop1 is compromised, there is a limiting amount of crossover designation potential such that one can only make the WT number of crossovers.

## Conclusions

Detailed analysis of meiotic prophase events in *Sordaria hop1* mutants has produced 3 important types of results. First, chromosome axes in some regions of the chromosomes are intrinsically prone to modulation during their transit from the leptotene coalignment state to the SC state. This is manifested specifically in the *hop1Δ* phenotype by: (i) the loss of Spo76/Pds5; (ii) a tendency for loss of Hop1 itself; (iii) failure to form SC all along homologs, and failure of recombination interactions to further develop. The same tendency might account for the fact that, in several other organisms, Hop1 is differentially lost from the chromosomes at pachytene. Analogous phenotypes occur in absence of Rec8 and in the *spo76-1* mutant. Thus, all 3 axis components appear to collaborate in some critical step which we suggest to be emergence of inter-axis bridges. Molecular details and mechanisms involved in these effects remain to be defined.

Second, aside the above-defined effects, Hop1 is revealed to have direct roles in several basic processes. It is required: (i) to give normal axis lengths; (ii) for correct homolog pairing, likely due to its involvement in spatial juxtaposition of axes; and, (iii) separately, it is also required for resolution of interlocks. Finally, Hop1 is shown to play a direct role in spreading of the crossover interference signal.

Third, non-null mutant phenotypes showed that WT crossover level can be maintained despite increased axis length and irrespective of homolog pairing/synapsis. They thus confirm and extend previous indications that the number of crossovers that form during meiosis is kept at a relatively constant level by effects that affect crossover designation.

## Methods

### Cloning and transformation of *Sordaria*

The null *hop1Δ* mutant was obtained by single-step gene replacement: a hygromycin-resistance cassette replaces the entire ORF. Transformants carrying a null allele were selected for hygromycin resistance and the presence of the deleted allele confirmed by DNA sequencing. Transformations were performed in a *ku70Δ* mutant background, which increases the homologous integration events. Further crosses with a *KU70* WT strain eliminated the *ku70Δ* allele. Introduction of an ectopic WT *HOP1* gene into the null mutant restored a WT phenotype.

GFP and mCherry coding sequences (p-EGFP-1, Clontech; pRsetB-mCherry) were fused to the C-terminus of *HOP1* under the control of the *HOP1* promoter. After validation by sequencing, the mCherry allele was ectopically integrated into both WT and *hop1Δ*. The Hop1-GFP allele was integrated into the *HOP1* locus by homologous recombination. Hop1-GFP and Hop1-mCherry strains were WT by all criteria and thus fully functional. Point mutants as well as domain-deletion mutants were created by PCR-based mutagenesis (from the HOP1-mCheery plasmid) and introduced in WT by co-transformation with a plasmid encoding the hygromycin resistance cassette.

### Homolog identification, alignments, phylogeny, and models

Hop1, Mad2, and Rev7 homologs were identified from series of PSI-BLAST and HHMER analyses at the Max Planck Institute (MPI) using the MPI Toolkit (https://toolkit.tuebingen.mpg.de). Multiple sequence alignments (MSAs) generated by MAFFT 7.0 (http://mafft.cbrc.jp/alignment/server) with the auto mode and default parameters were used as inputs. MSA inputs included only previously validated homologs (as described hereafter), and alternative query sequences were systematically used as the MSA header sequence to improve detection of remote homologs. Candidate proteins were validated or not by secondary structure prediction with PSI-PRED (http://bioinf.cs.ucl.ac.uk/psipred). Protein modelling was done with an advanced version of AlphaFold2 from ColabFold (https://github.com/sokrypton/ColabFold). The per-residue confidence score (pLDDT; values greater than 90 indicating high confidence) and distance error for every pair of residues (PAE) were provided by the ColabFold website. Modelled 3D structures were compared with PDBeFold (https://www.ebi.ac.uk/msd-srv/ssm/). Atomic structures were superimposed with MatchMaker in CHIMERA 1.16 (https://www.cgl.ucsf.edu/chimera/). Phylogenetic relationships were established with IQTree (http://iqtree.cibiv.univie.ac.at/). The consensus tree shown in S1A Fig resulted from 1,000 bootstrap trees and was inferred from the LG+F+R3 best-fit model of amino-acid substitution.

### RT-qPCR experiment

cDNA and RT-qPCR were performed as described in [25]. Cultures for RNA preparations were performed at 25°C from day 1 to day 9. RNAs were extracted from fruiting bodies, quantified and checked for integrity on a gel. Total RNAs were reverse transcribed with SuperScript III (Lifes Technologies). A bilateral Student test for heteroscedastic samples was used to compare the biological replicates obtained at day 1 with those of the other days. The cutoff for significant *p*-value was set below 0.01. RT-qPCR experiments were MIQE compliants.

### Focus distances and interference measurements

For Gamma and CoC analyses of crossover interference, inter Hei10 focus distances were measured from the center of the first focus to the center of the next focus, along all 7 chromosomes of *Sordaria* mid-pachytene nuclei (or along the SC segments seen in absence of Hop1), using public domain software ImageJ (details in [49]).

CO interference level by gamma distribution. To calculate the shape parameter (**ν**) of gamma distribution used to describe CO interference (increased **ν** indicates higher interference), the inter-adjacent-Hei10 foci distances were calculated as percentages of axis length. The best-fit gamma parameters for inter-Hei10 foci distances from all chromosomes were estimated by the maximum likelihood method with the “gamfit” function in MATLAB.

CO interference by the CoC. CoC analysis is the classic way to describe crossover interference because it directly indicates the extent to which COs in 2 intervals do or not occur independently. To do so: (i) we established a data set in which Inter-Hei10 focus distances were measured from the center of the first focus to the center of the next focus, along all chromosomes in each nucleus of each analyzed strain. (ii) Divided each bivalent into a number of intervals of equal size (roughly 5 times the average number of COs in that bivalent). (iii) On each bivalent, each CO was assigned to a specific interval. (iv) The frequency of bivalents with a CO in each given interval was calculated. (v) Intervals were then considered in all pairwise combinations. (vi) Each pair of intervals was then analyzed with respect to the frequency of the bivalents with COs in both intervals (“observed” double COs, given by the data sets) and, assuming COs occur independently in each interval, the frequency of “expected” double COs, given by the product of the observed CO frequencies in the 2 intervals considered separately: CoC is the quotient of observed/expected double COs. (vii) The CoCs for all pairs of intervals were then plotted, for each pair, as a function of the distances between the 2 intervals, providing a CoC curve. In a CoC curve, at short inter-interval distances the CoC values are very low which indicates that CO interference is strong. And with increasing inter-interval distances the CoC values increase and eventually CoC values fluctuate around one, which indicates there is no interference at this distance (thus independent occurrence of COs in the 2 intervals). The inter-interval distance at CoC = 0.5 was chosen as a good indicator for CO interference strength.

### Data analysis and statistics

Statistical analyses were performed in GraphPad Prism version 10.2 (GraphPad Software). Data were analyzed for statistical significance using unpaired two-tailed Student’s *t* test (Figs 2H, 2I, 3F, 3G, 4G,4H and 5G), Welch and Brown–Forsythe one-way analysis of variance (ANOVA) along with Dunn’s test (Figs 6C, 6D, 8A and 8E). Error bars indicate the standard error (SD), mean and error bar (SD) are indicated for each set. In the text, the datasets are represented as the mean ± SD. Significance values are indicated as follows: ns (not significant) *P* > 0.05, * *P* < 0.05, ** *P* < 0.01, *** *P* < 0.001, and **** *P* < 0,0001.

### Cytology

GFP, mCherry, Tomato-red, and DAPI (0.5 g/ml) signals were observed after fixation in 4% paraformaldehyde, with either a Zeiss Axioplan microscope with a CCD Princeton camera, a Leica THUNDER Imager 3D Tissue with a Leica K5 Camera or a Delta Vision OMXTM platform (3D-SIM; Applied Precision). Public domain software ImageJ (http://rsb.info.nih.gov/ij)) was used to deconvolute Z-series and treat the images.

## Supporting information

S1 Fig Phylogeny and predicted structure of the *Sordaria* Hop1 protein. (**A**) Phylogenetic relationships among HORMA domain proteins in diverse eukaryotes: *M*. *musculus* (MMUS), *C*. *elegans* (CELE), *S*. *cerevisiae* (SCER), *S*. *pombe* (SPOM), *A*. *thaliana* (ATHA), *A*. *nidulans* (ANID), and *S*. *macrospora* (SMAC). A consensus tree constructed from 1,000 bootstrap trees is shown with bootstrap supports (%). Branch lengths have been optimized by maximum likelihood on the original alignment. (**B**) AF2 modelled SMAC_06964/Hop1 protein. Rainbow coloring is shown from N- to C-termini as indicated (top); (bottom) model confidence with predicted per-residue local distance difference test (pLDDT; left) and alignment error for every pair of residues (PAE, right).
(TIF)

S2 Fig Expression kinetics, sporulation, and meiotic progression. (**A**) Time-course expression of *HOP1* versus *SPO11* and *MER2* genes during *Sordaria* sexual cycle. Day 1 corresponds to the vegetative cycle: only vegetative mycelium on the plates. At day 2: protoperithecia develop on mycelium, indicating the start of the sexual cycle. At days 3 and 4: fruiting bodies (perithecia) contain young asci (meiocytes) in meiotic prophase I. At days 5 and 6: all perithecia contain asci at different steps of meiosis. Days 7 to 9: most asci of the perithecia contain ascospores. Fold changes and 95% confidence interval (CI) are indicated. Fold changes are expressed relative to day 1. *SPO11* at day 2, and all genes after day 2 have a fold change with a *p*-value < 0.05. Exact *p*-values and data necessary for the construction of the figure are shown in S1 Data. Error bars indicate 95% CI. (**B**) Percentage of 8-spored asci in WT and the analyzed mutants. Each perithecium contains 80–100 asci and for each strain we analyzed the asci from 15–20 perithecia. Left top: Like all mutant perithecia, those of *hop1Δ+SCDΔ* contain a mixture of asci with 8 black (or gray if not mature) ascospores and asci with either 1–4 abnormal, more or less “empty” ascospores (red arrows) or asci with 8 aborted ascospores (green arrows), indication of a meiotic defect, in contrast with wild-type (WT) perithecia (left bottom) which exhibit 94% of 8-spored asci. Right: Histograms of the percentage of asci with 8-spored asci in each analyzed strain. (**C**) Meiotic-prophase progression in *Sordaria*. Left and middle, examples of WT asci at pre-karyogamy (PK), karyogamy (K), early leptotene (EL), mid-leptotene (ML), late Leptotene (LL), and Pachytene (P) with colocalization of Spo76-Tred and Hop1-GFP (left) and Spo76-Tred and Sme4-GFP (right). Sizes of the asci in microns: leptotene: 15 to 40, zygotene: 40 to 50, pachytene: 50 to 110 and metaphase-anaphase I: 120–130. Right: Note that the three asci of *hop1Δ* (colocalization of Spo76-Tred and Sme4-GFP) show the same ascus size progression than the WT asci. Scale bars: 5 μm. The raw data underlying panels S2A and S2B are available in S1 Data.
(TIF)

S3 Fig Localization of Spo76/Pds5, Sme4/Zip1, Rec8, and Hop1 in single and double mutants. (**A**) Sme4-GFP is visible as short segments in both WT (left) and in *hop1Δ* (right). Note the difference in chromatin diffuseness (DAPI) between WT and mutant. (**B**) In *hop1Δ*, at the post-pachytene diffuse stage, Ecm11-GFP forms short segments which contain Hei10 foci (arrow). (**C**) Localization of Spo76-GFP in single *spo11Δ* (left) and in double *hop1Δ spo11Δ* mutants. Note that Spo76 remains along the axes and chromatin is less diffuse than in the single *hop1Δ* mutant (S3A). (**D**) Localization of Spo76-GFP in single *sme4Δ* (left) and in double *hop1Δ sme4Δ* mutants. Spo76-GFP is visible along the coaligned axes of the 2 middle and right *hop1Δ sme4Δ* nuclei like in the single *sme4Δ* mutant. Corresponding DAPI, right. (**E**) Two examples of Sme4-GFP and Spo76-Tred (Tr) colocalization during mid-pachytene in *hop1Δ*. (**F**) Rec8-GFP and Sp76-Tred colocalize perfectly during *hop1Δ* leptotene (this nucleus is more flattened to better show colocalization). (**G**) In absence of Rec8, from mid-pachytene on, Hop1-GFP staining is always dotty, in contrast with the smoother staining seen earlier (Fig 4C). (**H**) Although less pronounced the same change in localization is also visible with Hop1-GFP in spo76-1. Note also the diffuseness of DAPI in this mutant. Right, merge of Hop1 and DAPI; nu = nucleolus. Scale bars: 2 μm.
(TIF)

S4 Fig Sme4, Zip4, Zip2, and Msh4 localizations in *hop1Δ* and haploid meiosis. (**A**) The *spo76-1* mutant being defective in sister-chromatid cohesion, only few SC segments are formed *per* nucleus, as confirmed here by Sme4-GFP. Middle, DAPI and merge, right. (**B–F**) **Haploid meiosis** in absence of Slp1. In the *slp1Δ* mutant, the 2 twin-haploid nuclei remain side by side and do never fuse. (**B–D**) Spo76-GFP. (**B**) At what should be pachytene by ascus size, Spo76-GFP forms 7 lines (left), which correspond to the DAPI signal (right). (**C**) The same is true in the double *hop1Δ slp1Δ* mutant at leptotene. (**D**) At pachytene, however, the Spo76 signal shows discontinuities (arrows), confirmed with DAPI colocalization (red in merge). (**E, F**) Ecm11-GFP and Hei10-GFP at pachytene. (**E**) In the single *slp1* mutant, SCs are formed between the sister chromatids as indicated by the presence of Ecm11-GFP and Hei10 foci (arrows) along the 7 chromosomes (drawn right). (**F**) In the *hop1Δ slp1Δ* double mutant, Ecm11-GFP lines with Hei10 foci (arrows) are also visible along chromosomes but lines are shorter and more numerous than in the single mutant (see drawing); corresponding DAPI, right. (**G, H**) In absence of Hop1, both Zip4 (**G**) and Zip2 (**H**) foci are visible at leptotene along chromosome axes, especially evident at the coalignment stage (merge with DAPI red, right). (**I**) Two pachytene nuclei showing that in absence of Hop1, Msh4 remains throughout pachytene as rows of foci, likely along the synapsed regions. Scale bars: 2 μm.
(TIF)

S5 Fig SC/crossover correlation, Rec8 localization, and diplotene/metaphase I in *hop1*Δ. (**A**) Linear regression analysis between the number of Hei10 foci per nucleus and the SC lengths in microns in wild type (orange) and *hop1Δ* (blue). Both coefficient of determination (R^2^) and the regression line are indicated in the graph. (**B**) Two *hop1Δ* pachytene nuclei. Rec8-GFP does not allow to follow each bivalent because chromosome axes are intermingled in the middle regions of the nuclei, but colocalization with Hei10 foci, which are only formed on SCs, shows that ends are mostly synapsed (arrowheads). Some ends remain, however, un-synapsed (arrows in right nucleus). In left nucleus, arrow points to a Hei10 focus located on only 1 homolog. (**C**) DAPI of *hop1Δ* diplotene (left), Metaphase I (middle), and Anaphase I (right) nuclei. The presence of 7 bivalents (arrows) indicates that all DSBs were repaired. Metaphase I nuclei, however, exhibit either 7 bivalents (left nucleus) or 6 bivalents and 2 univalents (arrows in middle-right nucleus). Example of Anaphase I with one lagging chromosome (arrow), likely the consequence of precocious homolog separation. Scale bars: 2 μm. The raw data underlying panel S5A are available in S1 Data.
(TIF)

S6 Fig Non-null *HOP1* mutants. (**A**) Hop1-only-HORMA-mCherry (mC) in a *hop1Δ* background (left) vs. Hop1-mCherry in WT (right). In both strains, the protein is visible along chromosome axes at leptotene. (**B**) The 2 pachytene nuclei of the *hop1-CTD* mutant illustrate 2 findings: (i) Hop1-CTD-mC and Spo76-GFP colocalize in both synapsed and unsynapsed (arrow in left nucleus) regions. (ii) Interlockings (arrows) are present from mid-pachytene (left nucleus) to late pachytene (right nucleus). Corresponding drawings allow to follow the path of the 7 homologs. Note that axes are less stiff at late pachytene and that both nuclei exhibit Hei10 foci along all homologs. (**C**) Double *hop1-CTD mlh1Δ* mutant. Interlockings (arrows) are present from late zygotene (left nucleus) to mid-pachytene (right nucleus and corresponding drawing). (**D**) There is a perfect colocalization of Spo76-GFP with Hop1-K14R-mC (left nucleus) and Hop1-K259R-mC (right nucleus) from zygotene (left nucleus) to end pachytene (right nucleus). Hei10 foci (green) are visible along all homologs in the right nucleus corresponding to the colocalization of Hop1-K259R-mC with Spo76-GFP + Hei10-GFP. (**E**) Perfect colocalization of Hop1-SCD-mC (left) with Spo76-GFP (middle) and merge. (**F**) Crossover interference defined by CoC analysis for bivalents 1 and 2 in *hop1-K259R* pachytene nuclei in which some bivalents are completely synapsed (green curve) while others show more or less long non-synapsed regions (black curve). The shape of the 2 curves indicates that the strength of interference is similar in the 2 types of bivalents. Scale bars: 2 μm. The raw data underlying panel S6F are available in S1 Data.
(TIF)

S1 Data Excel file with all individual numerical values corresponding to the data presented in the main and supplemental figures (corresponding figure numbers are indicated in each Excel worksheet). (XLSX)

## Abbreviations

3D-SIM: three-dimensional structured illumination microscopy
CM: closure motif
CoC: coefficient of coincidence
CTD: C-terminal domain
DSB: double-strand break
MSA: multiple sequence alignment
RMSD: root mean square deviation
SC: synaptonemal complex
SCD: S/TJQ cluster domain
WT: wild-type
