# Supplementary figures and images for: Canonical and noncanonical roles of Hop1 are crucial for meiotic prophase in the fungus Sordaria macrospora
Source: PLoS Biol. 2024 Jul 1;22(7):e3002705. doi: 10.1371/journal.pbio.3002705 (PMC11244814; doi:10.1371/journal.pbio.3002705)

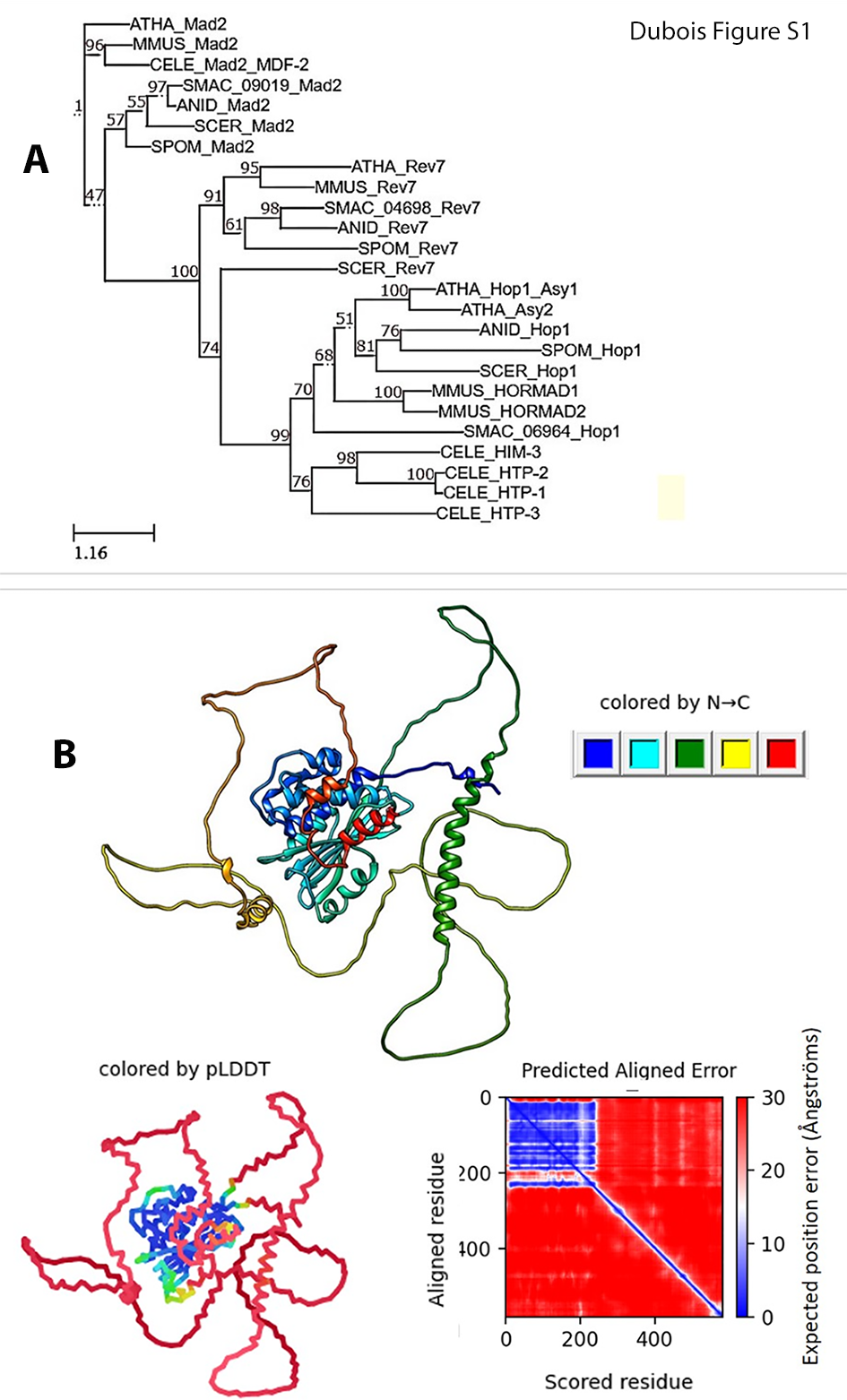

Supplement: S1 Fig — (A) Phylogenetic relationships among HORMA domain proteins in diverse eukaryotes: M. musculus (MMUS), C. elegans (CELE), S. cerevisiae (SCER), S. pombe (SPOM), A. thaliana (ATHA), A. nidulans (ANID), and S. macrospora (SMAC). A consensus tree constructed from 1,000 bootstrap trees is shown with bootstrap supports (%). Branch lengths have been optimized by maximum likelihood on the original alignment. (B) AF2 modelled SMAC_06964/Hop1 protein. Rainbow coloring is shown from N- to C-termini as indicated (top); (bottom) model confidence with predicted per-residue local distance difference test (pLDDT; left) and alignment error for every pair of residues (PAE, right). (TIF) [file pbio.3002705.s001.tif]

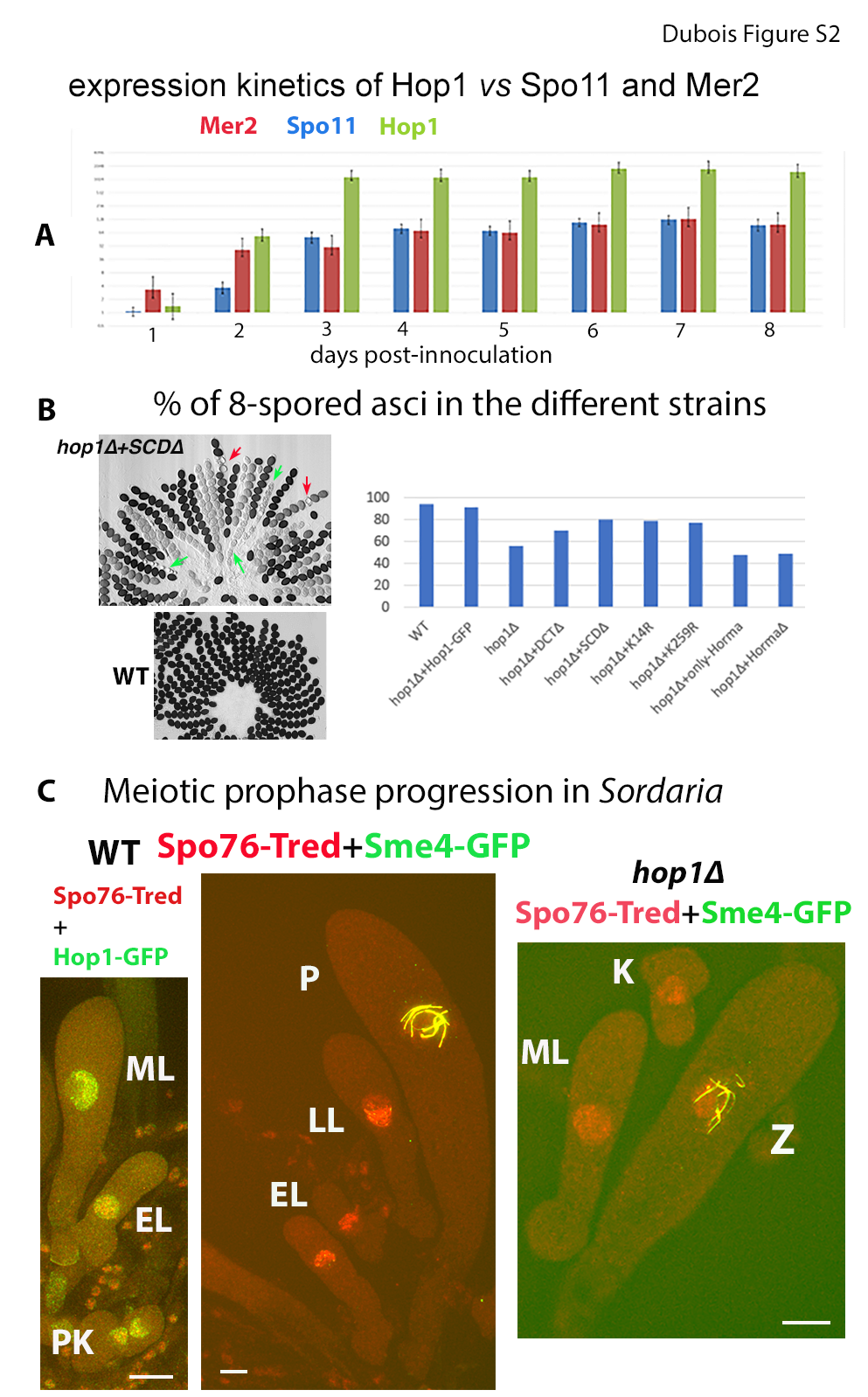

Supplement: S2 Fig — (A) Time-course expression of HOP1 versus SPO11 and MER2 genes during Sordaria sexual cycle. Day 1 corresponds to the vegetative cycle: only vegetative mycelium on the plates. At day 2: protoperithecia develop on mycelium, indicating the start of the sexual cycle. At days 3 and 4: fruiting bodies (perithecia) contain young asci (meiocytes) in meiotic prophase I. At days 5 and 6: all perithecia contain asci at different steps of meiosis. Days 7 to 9: most asci of the perithecia contain ascospores. Fold changes and 95% confidence interval (CI) are indicated. Fold changes are expressed relative to day 1. SPO11 at day 2, and all genes after day 2 have a fold change with a p-value < 0.05. Exact p-values and data necessary for the construction of the figure are shown in S1 Data. Error bars indicate 95% CI. (B) Percentage of 8-spored asci in WT and the analyzed mutants. Each perithecium contains 80–100 asci and for each strain we analyzed the asci from 15–20 perithecia. Left top: Like all mutant perithecia, those of hop1Δ+SCDΔ contain a mixture of asci with 8 black (or gray if not mature) ascospores and asci with either 1–4 abnormal, more or less “empty” ascospores (red arrows) or asci with 8 aborted ascospores (green arrows), indication of a meiotic defect, in contrast with wild-type (WT) perithecia (left bottom) which exhibit 94% of 8-spored asci. Right: Histograms of the percentage of asci with 8-spored asci in each analyzed strain. (C) Meiotic-prophase progression in Sordaria. Left and middle, examples of WT asci at pre-karyogamy (PK), karyogamy (K), early leptotene (EL), mid-leptotene (ML), late Leptotene (LL), and Pachytene (P) with colocalization of Spo76-Tred and Hop1-GFP (left) and Spo76-Tred and Sme4-GFP (right). Sizes of the asci in microns: leptotene: 15 to 40, zygotene: 40 to 50, pachytene: 50 to 110 and metaphase-anaphase I: 120–130. Right: Note that the three asci of hop1Δ (colocalization of Spo76-Tred and Sme4-GFP) show the same ascus size progression than [file pbio.3002705.s002.tif]

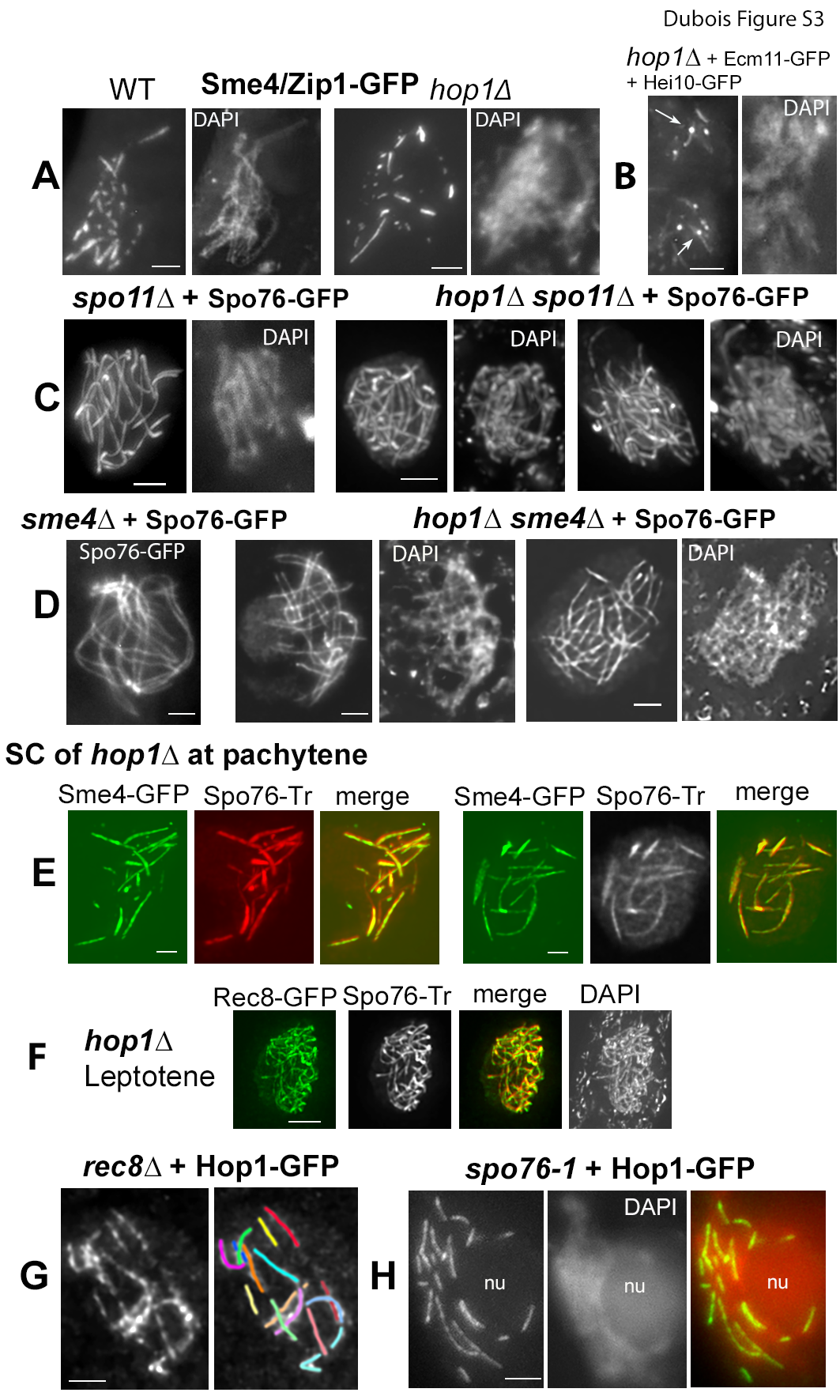

Supplement: S3 Fig — (A) Sme4-GFP is visible as short segments in both WT (left) and in hop1Δ (right). Note the difference in chromatin diffuseness (DAPI) between WT and mutant. (B) In hop1Δ, at the post-pachytene diffuse stage, Ecm11-GFP forms short segments which contain Hei10 foci (arrow). (C) Localization of Spo76-GFP in single spo11Δ (left) and in double hop1Δ spo11Δ mutants. Note that Spo76 remains along the axes and chromatin is less diffuse than in the single hop1Δ mutant (S3A). (D) Localization of Spo76-GFP in single sme4Δ (left) and in double hop1Δ sme4Δ mutants. Spo76-GFP is visible along the coaligned axes of the 2 middle and right hop1Δ sme4Δ nuclei like in the single sme4Δ mutant. Corresponding DAPI, right. (E) Two examples of Sme4-GFP and Spo76-Tred (Tr) colocalization during mid-pachytene in hop1Δ. (F) Rec8-GFP and Sp76-Tred colocalize perfectly during hop1Δ leptotene (this nucleus is more flattened to better show colocalization). (G) In absence of Rec8, from mid-pachytene on, Hop1-GFP staining is always dotty, in contrast with the smoother staining seen earlier (Fig 4C). (H) Although less pronounced the same change in localization is also visible with Hop1-GFP in spo76-1. Note also the diffuseness of DAPI in this mutant. Right, merge of Hop1 and DAPI; nu = nucleolus. Scale bars: 2 μm. (TIF) [file pbio.3002705.s003.tif]

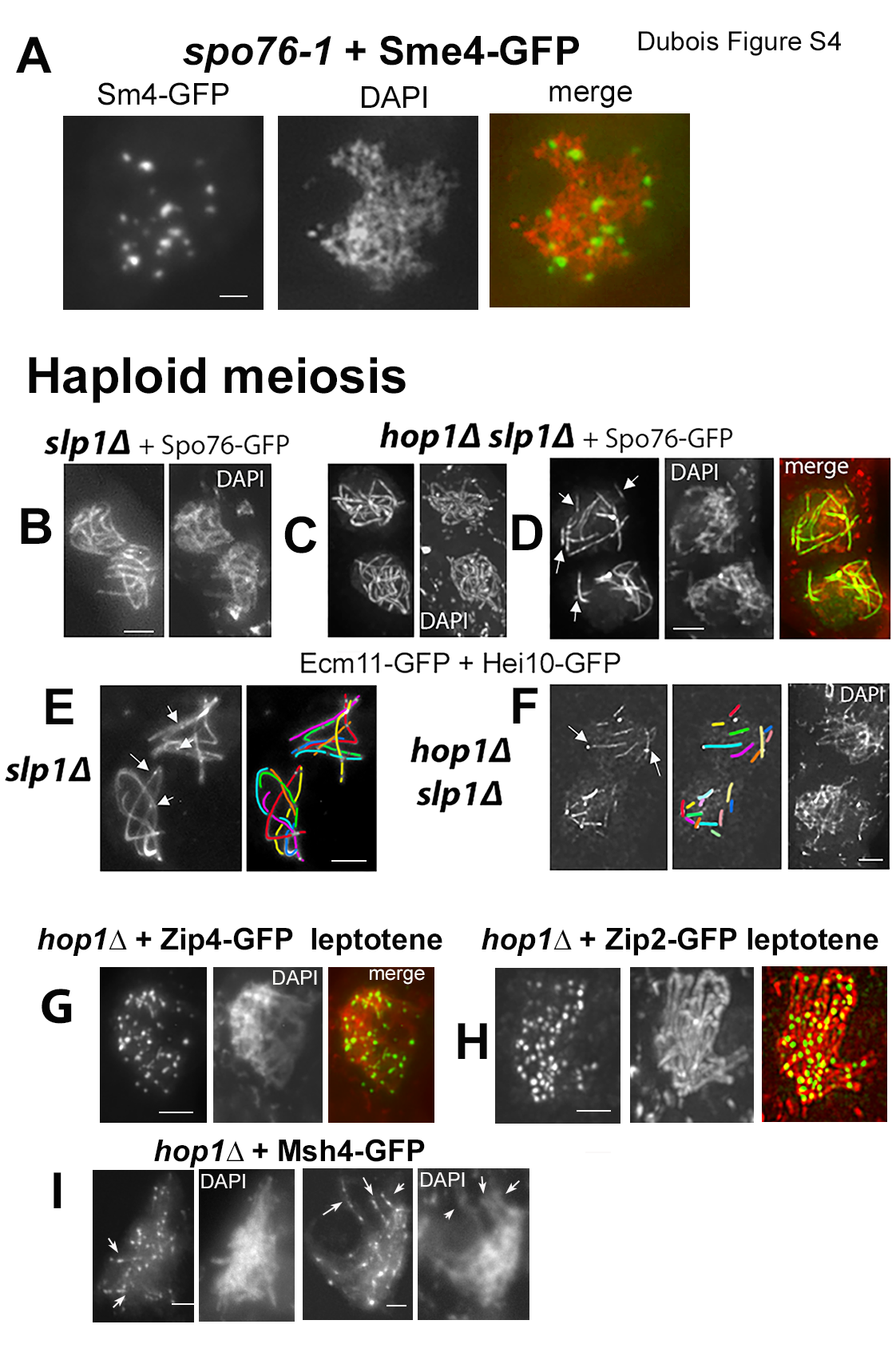

Supplement: S4 Fig — (A) The spo76-1 mutant being defective in sister-chromatid cohesion, only few SC segments are formed per nucleus, as confirmed here by Sme4-GFP. Middle, DAPI and merge, right. (B–F) Haploid meiosis in absence of Slp1. In the slp1Δ mutant, the 2 twin-haploid nuclei remain side by side and do never fuse. (B–D) Spo76-GFP. (B) At what should be pachytene by ascus size, Spo76-GFP forms 7 lines (left), which correspond to the DAPI signal (right). (C) The same is true in the double hop1Δ slp1Δ mutant at leptotene. (D) At pachytene, however, the Spo76 signal shows discontinuities (arrows), confirmed with DAPI colocalization (red in merge). (E, F) Ecm11-GFP and Hei10-GFP at pachytene. (E) In the single slp1 mutant, SCs are formed between the sister chromatids as indicated by the presence of Ecm11-GFP and Hei10 foci (arrows) along the 7 chromosomes (drawn right). (F) In the hop1Δ slp1Δ double mutant, Ecm11-GFP lines with Hei10 foci (arrows) are also visible along chromosomes but lines are shorter and more numerous than in the single mutant (see drawing); corresponding DAPI, right. (G, H) In absence of Hop1, both Zip4 (G) and Zip2 (H) foci are visible at leptotene along chromosome axes, especially evident at the coalignment stage (merge with DAPI red, right). (I) Two pachytene nuclei showing that in absence of Hop1, Msh4 remains throughout pachytene as rows of foci, likely along the synapsed regions. Scale bars: 2 μm. (TIF) [file pbio.3002705.s004.tif]

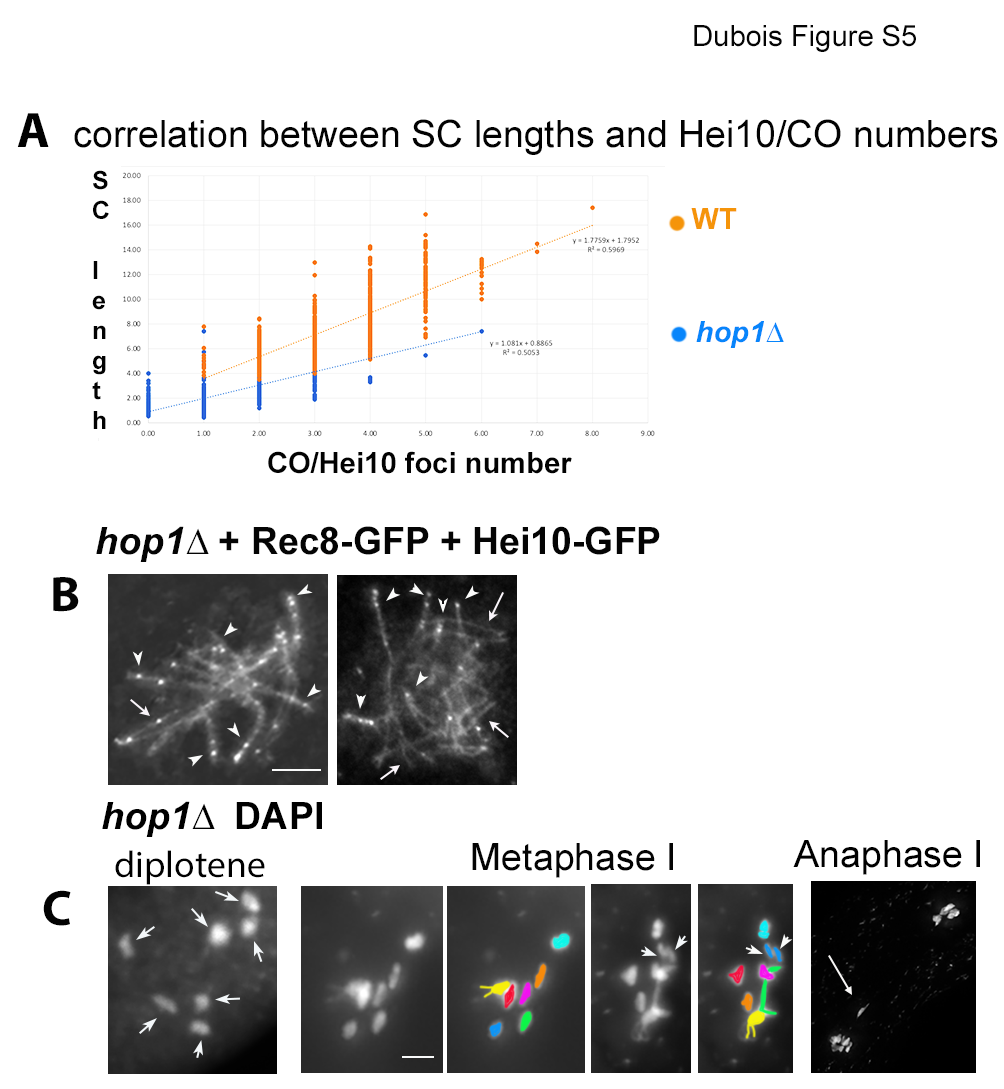

Supplement: S5 Fig — (A) Linear regression analysis between the number of Hei10 foci per nucleus and the SC lengths in microns in wild type (orange) and hop1Δ (blue). Both coefficient of determination (R2) and the regression line are indicated in the graph. (B) Two hop1Δ pachytene nuclei. Rec8-GFP does not allow to follow each bivalent because chromosome axes are intermingled in the middle regions of the nuclei, but colocalization with Hei10 foci, which are only formed on SCs, shows that ends are mostly synapsed (arrowheads). Some ends remain, however, un-synapsed (arrows in right nucleus). In left nucleus, arrow points to a Hei10 focus located on only 1 homolog. (C) DAPI of hop1Δ diplotene (left), Metaphase I (middle), and Anaphase I (right) nuclei. The presence of 7 bivalents (arrows) indicates that all DSBs were repaired. Metaphase I nuclei, however, exhibit either 7 bivalents (left nucleus) or 6 bivalents and 2 univalents (arrows in middle-right nucleus). Example of Anaphase I with one lagging chromosome (arrow), likely the consequence of precocious homolog separation. Scale bars: 2 μm. The raw data underlying panel S5A are available in S1 Data. (TIF) [file pbio.3002705.s005.tif]

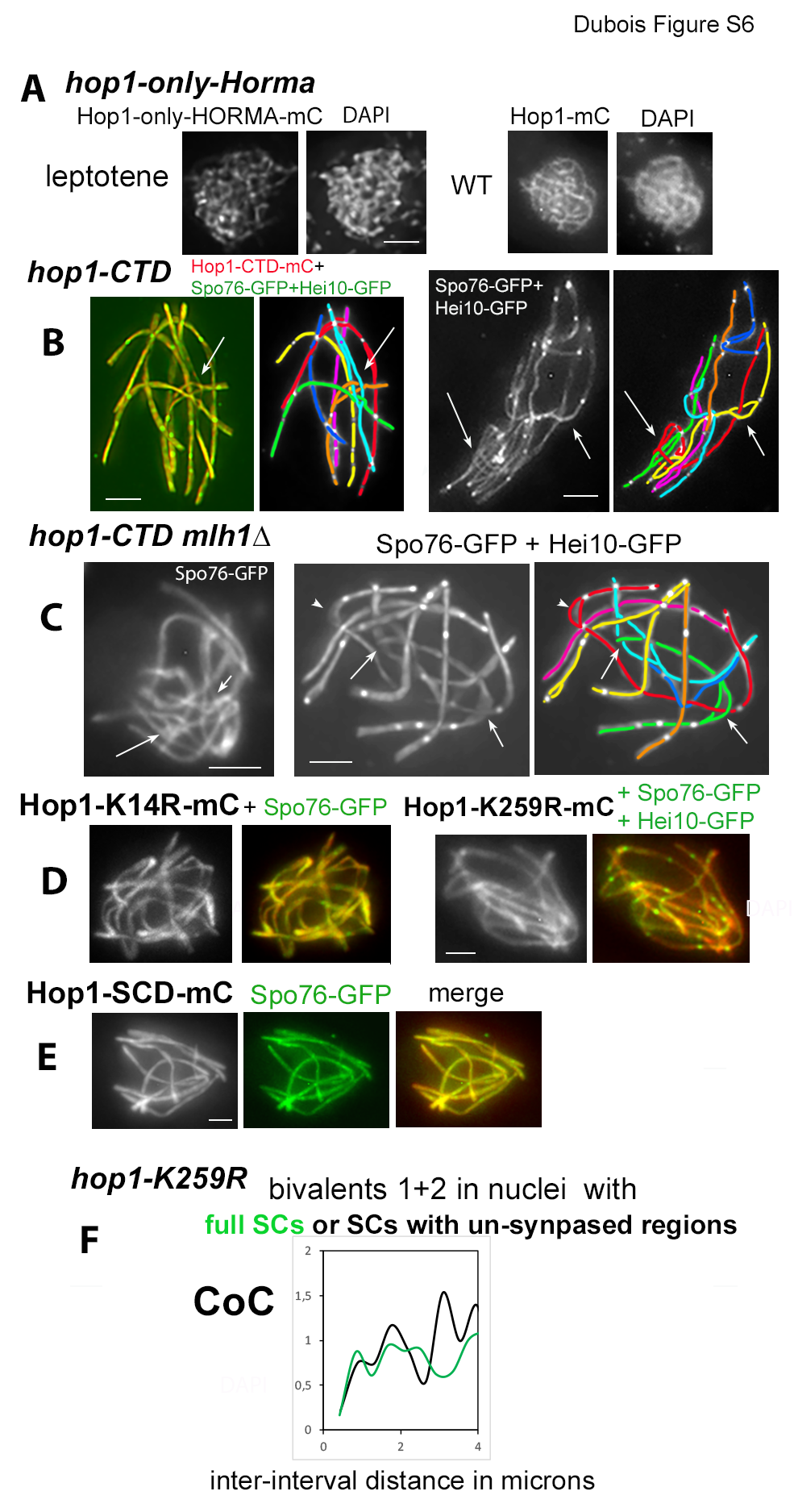

Supplement: S6 Fig — (A) Hop1-only-HORMA-mCherry (mC) in a hop1Δ background (left) vs. Hop1-mCherry in WT (right). In both strains, the protein is visible along chromosome axes at leptotene. (B) The 2 pachytene nuclei of the hop1-CTD mutant illustrate 2 findings: (i) Hop1-CTD-mC and Spo76-GFP colocalize in both synapsed and unsynapsed (arrow in left nucleus) regions. (ii) Interlockings (arrows) are present from mid-pachytene (left nucleus) to late pachytene (right nucleus). Corresponding drawings allow to follow the path of the 7 homologs. Note that axes are less stiff at late pachytene and that both nuclei exhibit Hei10 foci along all homologs. (C) Double hop1-CTD mlh1Δ mutant. Interlockings (arrows) are present from late zygotene (left nucleus) to mid-pachytene (right nucleus and corresponding drawing). (D) There is a perfect colocalization of Spo76-GFP with Hop1-K14R-mC (left nucleus) and Hop1-K259R-mC (right nucleus) from zygotene (left nucleus) to end pachytene (right nucleus). Hei10 foci (green) are visible along all homologs in the right nucleus corresponding to the colocalization of Hop1-K259R-mC with Spo76-GFP + Hei10-GFP. (E) Perfect colocalization of Hop1-SCD-mC (left) with Spo76-GFP (middle) and merge. (F) Crossover interference defined by CoC analysis for bivalents 1 and 2 in hop1-K259R pachytene nuclei in which some bivalents are completely synapsed (green curve) while others show more or less long non-synapsed regions (black curve). The shape of the 2 curves indicates that the strength of interference is similar in the 2 types of bivalents. Scale bars: 2 μm. The raw data underlying panel S6F are available in S1 Data. (TIF) [file pbio.3002705.s006.tif]
